# Fatty acid binding to the human transport proteins FABP3, FABP4, and FABP5 from a Ligand’s perspective

**DOI:** 10.1016/j.jbc.2024.107396

**Published:** 2024-05-20

**Authors:** Sebastian Michler, Florian Arndt Schöffmann, Dina Robaa, Jonas Volmer, Dariush Hinderberger

**Affiliations:** 1 Physical Chemistry – Complex Self-Organizing Systems, Institute of Chemistry, Martin Luther University Halle-Wittenberg, Halle (Saale), Germany; 2 Department of Medicinal Chemistry, Institute of Pharmacy, Martin Luther University Halle-Wittenberg, Halle (Saale), Germany

**Keywords:** fatty acid binding protein, microscale thermophoresis, dynamic light scattering, electron paramagnetic resonance (EPR), protein aggregation, protein dynamics, thermodynamics, transition-state analog

## Abstract

Fatty acid binding proteins (FABPs) are a family of amphiphilic transport proteins with high diversity in terms of their amino acid sequences and binding preferences. Beyond their main biological role as cytosolic fatty acid transporters, many aspects regarding their binding mechanism and functional specializations in human cells remain unclear. In this work, the binding properties and thermodynamics of FABP3, FABP4, and FABP5 were analyzed under various physical conditions. For this purpose, the FABPs were loaded with fatty acids bearing fluorescence or spin probes as model ligands, comparing their binding affinities *via* microscale thermophoresis (MST) and continuous-wave electron paramagnetic resonance (CW EPR) spectroscopy. The CW EPR spectra of non-covalently bound 5- and 16-DOXYL stearic acid (5/16-DSA) deliver in-depth information about the dynamics and chemical environments of ligands inside the binding pockets of the FABPs. EPR spectral simulations allow the construction of binding curves, revealing two different binding states (‘intermediately’ and ‘strongly’ bound). The proportion of bound 5/16-DSA depends strongly on the FABP concentration and the temperature but with remarkable differences between the three isoforms. Additionally, the more dynamic state (‘intermediately bound’) seems to dominate at body temperature with thermodynamic preference. The ligand binding studies were supplemented by aggregation studies *via* dynamic light scattering and bioinformatic analyses. Beyond the remarkably fine-tuned binding properties exhibited by each FABP, which were discernible with our EPR-centered approach, the results of this work attest to the power of simple spectroscopic experiments to provide new insights into the ligand binding mechanisms of proteins in general on a molecular level.

Fatty acids and other amphiphilic or even hydrophobic molecules need to transit various hydrophilic environments within the human body. This thermodynamically unfavorable pathway gave rise to the evolution of amphiphilic transport proteins. In the cytosol of vertebrate cells, this role is fulfilled by the fatty acid binding proteins (FABPs) (1). FABPs represent a family of homologous β-barrel proteins with a molecular weight of 14 to 15 kDa (2). Due to their amphiphilic properties, FABPs are water-soluble and can non-covalently bind and transport hydrophobic molecules (3). However, one basic question has frequently been raised in the course of studies on FABPs: Why does nature encode multiple isoforms of FABPs by greatly varying amino acid sequences? Currently, 12 FABP isoforms are known which are expressed and utilized in different tissues (4, 5, 6). They differ in their primary sequences (only ∼51–65% sequence identity) but share an almost identical tertiary structure (1). While a convergent evolution of analogous proteins would theoretically be possible, it was proposed that all FABPs could have evolved from one ancestral gene (1). Accordingly, the strong sequence diversification while preserving the tertiary structure of the FABPs would likely be attributable to an evolutionary adaption. The requirements for functional and tissue-specific adaptions, as well as binding specializations in complex organisms or environmental variety in the availability of amino acids, may contribute to an answer to the phenomenon of tertiary structure identity and primary sequence diversity (1). Within the scope of this work, we therefore compared the nanoscopic binding properties and their thermodynamics of FABP3, FABP4, and FABP5 from the perspective of their ligands (see Fig. 1).

**Figure 1:**
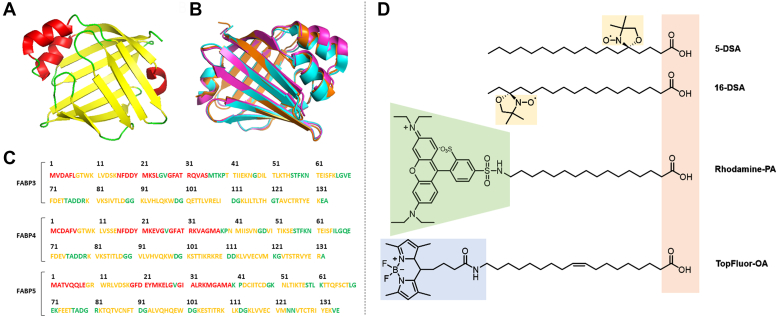
**Overview of the FABPs and ligands.***A*, crystal structure of apo-FABP3 (48), colored by secondary structural elements (*red*: α-helices, *yellow*: β-sheets, *green*: β-turns); the design is inspired by Armstrong *et al.* (50). *B*, aligned crystal structures of FABP3 (*violet*), FABP4 (*blue*) (13) and FABP5 (*orange*) (50). *C*, amino acid sequences of the FABPs with the same color code for secondary structural elements as in (*A*). *D*, chemical structures of the spin probes 5/16-DSA and the fluorophores rhodamine-PA, excited by *green light*, and TopFluor-OA, excited by *blue light*. The carboxylic acid headgroups are shown in *red* and the DOXYL groups in *yellow*.

The structural properties of the FABPs determine their biological functions. The three FABPs in focus share relatively low amino acid sequence similarities of 65% between FABP3 and FABP4, 51% between FABP3 and FABP5, and 55% between FABP4 and FABP5 (Fig. 1*C*). Despite these differences, their crystal structures are almost identical as shown in the alignment in Figure 1*B*. FABPs consist of ten antiparallel β-strands, organized in two orthogonal, five-stranded β-sheets (Fig. 1*A*) (1). Together they form a closed β-barrel structure, with an additional helix-turn-helix motif and a 3_10_-Helix at the N-terminus. It is proposed that the helix-turn-helix motif serves as a portal region for entering ligands, while the inner hydrophobic β-barrel forms a binding cavity for the ligand (7). While the extracellular transport protein human serum albumin (HSA) can bind at least up to seven ligands in binding pockets per molecule (8), only one ligand can be bound by one FABP molecule, except for FABP1 that can bind two ligands at once (9). All FABPs can bind and transport saturated and unsaturated long-chain fatty acids consisting of a hydrophilic carboxyl head group and a hydrophobic alkyl chain with at least 13 carbon atoms (10). As precursors and important constituents of lipids, these molecules do not only participate in cell membrane formation but also in storage, β-oxidation, signaling, and metabolic processes in cell organelles such as mitochondria and nuclei (4, 11). The FABP binding affinity for long-chain fatty acids is increased with higher hydrophobicity and hence longer fatty acid chains but is decreased with a higher number of unsaturated bonds (1). Furthermore, the affinities and dissociation constants (*K*_D_) significantly differ for certain FABP isoforms and fatty acids (2, 12). Various FABPs may also bind other hydrophobic ligands like eicosanoids, bile salts, peroxisome proliferators (1), and even synthetic drugs (13). Interactions with large macromolecules have also been reported, including proteins, enzymes, receptors, genes, as well as entire cell membranes (14). Consequently, FABPs are associated with a variety of biological functions and diseases.

FABP3 or heart-type fatty acid binding protein (H-FABP) is expressed primarily in the heart, skeletal muscle, and brain cells (15). There, FABP3 binds and transports fatty acids to the β-oxidation system in the mitochondria (11). Thus, the protein regulates the energy balance and the amount of adipose tissue. FABP3, along with FABP4, is also the major component of the mammary-derived growth inhibitor (MDGI) which leads to the assumption that FABP3 inhibits the growth of human breast cancer cells (16, 17). Finally, FABP3 is able to activate the peroxisome proliferator-activated receptor gamma (PPARγ) in the cell nuclei by delivering activator ligands to the nuclei (18). This function was also found for FABP4, which is also referred to as adipocyte-type fatty acid binding protein (A-FABP) (19). FABP4 is expressed in adipocytes and macrophages (20), as well as in dendritic cells (21). This FABP seems to play an important role in the development of metabolic syndrome, including an association with obesity, type II diabetes, cardiovascular diseases, and atherosclerosis (11). Finally, FABP4 modulates inflammatory responses and the accumulation of cholesterol esters in macrophages (11). The third isoform of this study, FABP5 or E-FABP is mainly expressed in epidermal cells. However, it is also present in smaller amounts in adipocytes and macrophages, as well as in the brain, kidney, liver, lung, and testis (21, 22, 23). Its occurrence alongside FABP4 seems to indicate at least partial functional overlap between these FABPs. This assumption is supported by the fact that FABP5 can be overexpressed in adipocytes when a loss of FABP4 occurs (11) and that FABP5 is correlated with the development of atherosclerosis and insulin resistance, too (24). FABP5 seems to be connected with the epidermal growth factor receptor (EGFR), which regulates endothelial cell proliferation, promoting the growth of tumor cells in breast and prostate cancer (25). This FABP also binds docosahexaenoic acid (DHA) with high affinity, as found by isothermal titration calorimetry (15, 26). Therefore, it possibly affects the DHA level in the brain. Finally, FABP5 is involved in skin homeostasis, affecting the development of the skin diseases psoriasis (27) and atopic dermatitis (28).

It is known that FABPs solubilize fatty acids similar to non-polar solvents by providing a hydrophobic microenvironment within their β-barrel (12, 29). However, important details about the binding and release mechanism remain unclear, *e.g.*, how differences in the sequences and molecular environments are possibly related to the binding selectivity of different FABP isomers. The experimental approach behind this work is based on the self-driven uptake and non-covalent binding of modified fatty acids by FABPs. The use of spectroscopically detectable (labeled) fatty acids enables an observation of the binding mechanism from the ligand’s perspective. For this task, CW EPR spectroscopy is well-suited. The EPR-active radicals 5- and 16-DSA are used as so-called spin probes. These nitroxide-labeled stearic acid molecules (see Fig. 1*D*) are co-dissolved with and then taken up by the FABPs. Their subsequently measured CW EPR spectra and spectral simulations provide information about the binding state, dynamics, and molecular environment of the ligands within the binding pocket. These properties can be obtained from the number and weighting of spectral components, the rotational correlation time τ_c,_ and the hyperfine coupling constant *a*, respectively. Comparable EPR-based approaches were frequently used for analyses of Serum Albumin (30, 31, 32, 33), and for FABP3 by Fournier *et al.* (34, 35), but not nearly to the extent as in this work. For example, isoform- and temperature-dependent binding studies as well as polarity studies on FABPs with CW EPR spectroscopy are for the first time described here. Besides using spin-labeled fatty acids, the FABPs can be also probed with fluorescent fatty acid variants. Here, [16-N-(lissamine rhodamine ß sulfonyl)amino] palmitic acid (rhodamine-PA) and [N-(4-TopFluor butanoyl) amino] oleic acid (TopFluor-OA) were selected for supplementing MST binding studies, in order to compare binding curves and dissociation constants (*K*_D_) of the three FABPs with this fluorescence-based detection method. MST binding studies with FABPs were recently done, *e.g.*, by Ayo *et al.* (36) who demonstrated successful binding between FABP3 and the glioblastoma-targeting polypeptide CooP with a *K*_D_ of 2.18 μM. Furthermore, Hofer *et al.* (37) utilized MST to prove the weak interaction between FABP4 and the enzyme activator Cgi-58, with a *K*_D_ value of 192 μM.

Beyond the binding of ligands, also the possibility of self-binding (aggregation) between FABPs was examined within the scope of this work. To this end, DLS has been applied to identify occurring dimers and aggregates and to analyze whether bound ligands influence this effect. In the following sections, the experimental details and results of our comparative binding studies on FABP3, FABP4, and FABP5 will be presented. Finally, possible correlations with bioinformatic analyses will be introduced and the results will be critically discussed in the context of the biological functions of FABPs.

## Results and discussion

### Binding of fatty acids depends on the sequence and concentration of FABPs

First, the concentration-dependent binding of fatty acids to FABP3, FABP4, and FABP5 (for brevity, we use FABPx to denote all three FABP isoforms, where adequate) was analyzed at a physiological temperature of 37 °C. For spectroscopic reasons, the concentrations of the fatty acids were left constant and only the FABP concentrations were changed. As a result, the binding process was analyzed from the perspective of the ligands, not from the macromolecule as it is mostly done.

### MST binding studies

For MST binding studies, 80 nM of the red fluorescent rhodamine palmitic acid (PA) was mixed with the concentration series of the FABPs. The MST-derived binding curves in Fig. S1 reveal that the lowest binding affinity occurs between FABP4 and rhodamine-PA (*K*_D_ = 10.5 ± 2.1 μM), closely followed by FABP5 (*K*_D_ = 8.3 ± 0.7 μM), while FABP3 shows the highest affinity (*K*_D_ = 3.5 ± 0.4 μM). All binding curves obtained for rhodamine-PA display relatively smooth and clearly sigmoidal shapes with tolerable error bars in comparison with the literature (36, 37). The *K*_D_ values are generally higher than those reported for non-labeled PA in literature, where values of 0.96 ± 0.05 μM, 2.86 ± 0.59 μM and 1.04 ± 0.16 μM have been obtained for FABP3, FABP4 and FABP5 by Scatchard plots (2, 38). These differences might occur due to the influence of the large rhodamine group which is covalently bound at position C16 of PA. The sterically demanding and positively charged fluorophore could hence influence, particularly decrease, the binding affinities. FABPs are known to bind more hydrophobic ligands with higher affinity (1). Since the polar fluorescent groups should increase the polarity of the fatty acids, they could be directly responsible for the increased *K*_D_ values. Nevertheless, the binding of fatty acids in general still seems to be relatively robust, and despite the occurrence of large polar groups, the labeled fatty acids are still bound. Moreover, the ranking of the binding affinities, increasing from FABP4 *via* FABP5 to FABP3, corresponds to that reported in the literature and the values are at a similar order of magnitude (2, 38), confirming the reliability of the values and the method MST. No binding curves could be obtained for the green fluorescent TopFluor-OA. This could either be due to the fact that the three FABPs are unable to bind this ligand in general, or that this ligand, especially containing the fluorescence dye, is inappropriate for the condition and setup used. The latter reason seems to be more plausible as it is known that FABPs are capable of binding oleic acid with moderate to high binding affinities (*K*_D_ ∼ 0.44–1.56 μM) (2).

From the binding affinities *K*_A_ (with *K*_A_ = *K*_D_^−1^), the molar Gibbs energies of association ${\Delta G}_{m,A}$ at 22 °C were calculated using the van’t Hoff equation (see below). The values are summarized in Table 1. The negative ΔG values signalize that the process is clearly exergonic in the direction of the association, as it was expected for ligand binding by transport proteins.

**Table 1:** Binding parameters for FABP3, FABP4 and FABP5 obtained by MST measurements

FABPx	*K*_D_ [μM]	*K*_A_ [μM^−1^]	*K*_A_ [M^−1^]	ln*K*_A_	Δ*G*_m,A_ [kJ mol^−1^]
FABP3	3.5 ± 0.4	0.286 ± 0.033	286,000 ± 33,000	12.56 ± 0.11	−30.8 ± 0.27
FABP4	10.5 ± 2.1	0.095 ± 0.019	95,000 ± 19,000	11.46 ± 0.18	−28.1 ± 0.44
FABP5	8.3 ± 0.7	0.120 ± 0.01	120,000 ± 10,000	11.7 ± 0.08	−28.7 ± 0.2

### Concentration-dependent CW EPR spectra and simulations

**Figure 2:**
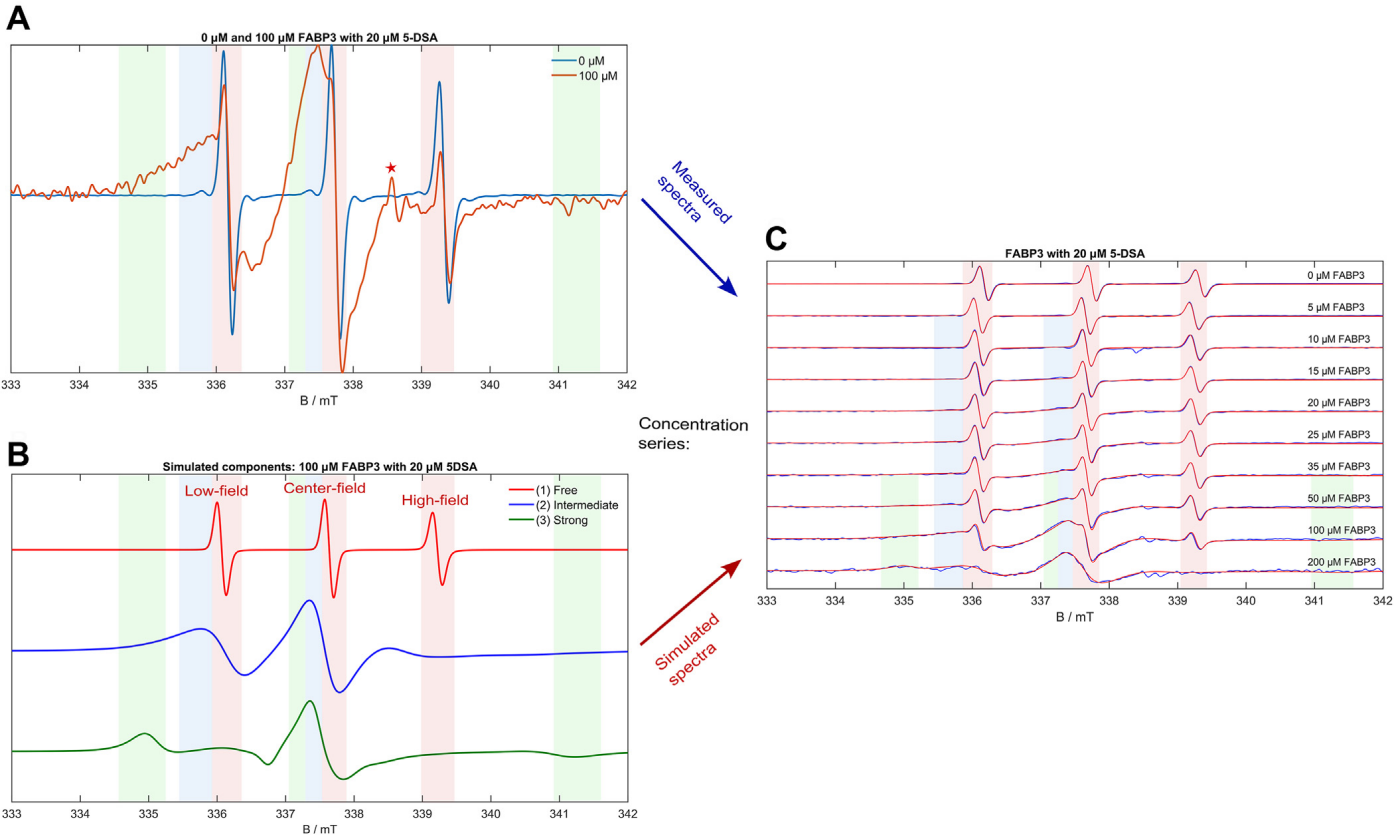
**CW EPR spectra (concentration series) of FABP3 with 5-DSA.***A*, spectra of 20 μM 5-DSA with 0 μM and 100 μM FABP3. *B*, simulated spectral components for 20 μM 5-DSA with 100 μM FABP3. *C*, complete concentration series with spectra (*blue*) and simulations (*red*) of FABP3 with 20 μM 5-DSA. Corresponding measurement series for FABP4 and FABP5 with 5- and 16-DSA are summarized in the SI (Figs. S3 and S4).

For a more in-depth view of the binding mechanism, CW EPR spectra of 5/16-DSA with concentration series of the FABPs were measured and simulated. This is shown in Figure 2 for FABP3 with 5-DSA, the remaining spectra and simulation series can be found in Figs. S3 and S4. Equal concentrations of 20 μM of each spin probe (5- and 16-DSA) were mixed with FABP concentrations of 0 to 200 μM, increasing the resulting ligand/protein ratio in the samples stepwise from 1/0 to 1/10.

Figure 2*A* shows the CW EPR spectra of pure 20 μM 5-DSA (blue spectrum) and 20 μM 5-DSA mixed with the fivefold amount of FABP3 (red spectrum). The blue-line spectrum represents a typical nitroxide CW EPR spectrum of rapidly and freely (isotropically) rotating 5-DSA molecules in solution, consisting of sharp low-, center- and high-field peaks. They are highlighted through red-striped regions. However, in the red-line spectrum from the same spin probe concentration with 100 μM FABP3, additional slower rotating spectral components appear, marked by green and blue striped regions. These are interpreted as stemming from 5-DSA molecules bound to FABP3, which makes their rotational motion anisotropic and severely slowed down (the quantification follows below). As shown in Figure 2*C*, the relative contribution of these bound components increases continually with increasing protein/ligand ratio for both spin probes with all FABPs, with the exception in the case of FABP4 with 5-DSA, where a concentration increase from 50 to 100 μM FABP4 leads to a jump-like change.

To analyze the spectra quantitatively, subsequent EPR spectral simulations were performed (Fig. 2, *B* and *C*). Three overlaying spectral components with different rotational correlation times τ_c_ were simulated, *e.g.*, (1), with 0.1 ns (red line) (2), with 4.1 ns (blue line), and (3) with 17.8 ns (green line) for the system 5-DSA/FABP3 (Fig. 2*B*). For a better understanding, a first interpretation of these components is given at this point. The rotational dynamics are thought to be indirectly proportional to the strength of intermolecular interactions between fatty acid and FABP. Therefore, they are indicative of the binding state of the fatty acids. Higher rotational correlation times signalize lower ligand dynamics and stronger interactions with the protein. Therefore, component (1) is interpreted as free (2), as intermediately (‘loosely’) bound, and (3) as strongly bound 5/16-DSA. Comparable spectral components with similar interpretations were already simulated, *e.g.*, for 16-DSA with Serum Albumin (31, 32). In the previously published CW EPR experiments with 12-DSA and porcine FABP3, performed by Fournier *et al.* (35), only one bound component could be identified. The three components occur for all FABP/spin probe systems, with the slightly varying parameters summarized in Tables S1–S3. A possible interpretation of two distinguishable, bound components is the occurrence of two thermodynamic binding states. We assume that the fatty acids can be either loosely attached to the protein surface or to the portal region of the β-barrel, which would constitute the intermediately bound species. The strongly bound component may be interpreted as being bound within the binding pocket *via* polar and non-polar amino acid residues. An alternative interpretation of the strongly bound component including self-aggregation and a more in-depth discussion of the bound components with the biological context is given below. The simulated slight Heisenberg exchange broadening (spin-spin interaction) of intermediately bound 5/16-DSA molecules leads to slightly broader spectra and rather indicates higher “local” concentrations, *i.e.*, few 5/16-DSA molecules being in a relatively close distance to each other. This exchange broadening can only occur if radicals are located very close distance each other, colliding with each other, leading to partial overlap of spin-bearing orbitals. The observed exchange kinetics could therefore argue for a dynamic exchange of ligands with temporarily close contacts. If the molecules are attached to the portal region of the β-barrel, for example, it is conceivable that they compete with each other for the interaction with surface-exposed amino acid residues.

**Figure 3:**
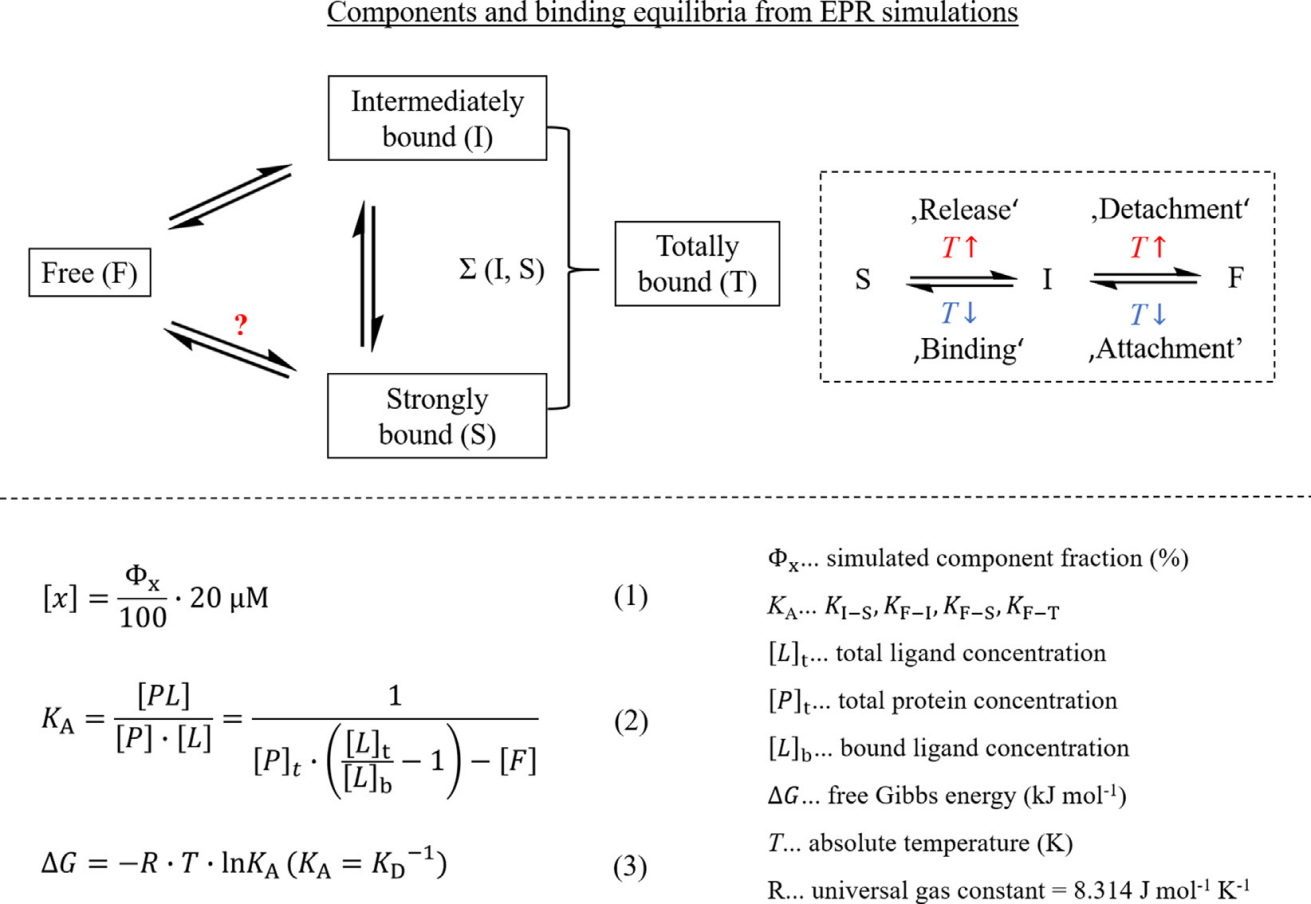
**Overview of the binding equilibria for the analyzed systems and equations (1) to (4) for the calculation of concentrations [*x*], equilibrium association/dissociation constants *K***_**A**_**and Gibbs energies Δ*G***.

A schematic view of the possible equilibria between all the states in the systems is shown in Figure 3, alongside different equations that will become important for the following quantitative analysis of the binding equilibria. This includes the before-mentioned van’t Hoff equation, Eq. (3), for the calculation of Gibbs energies.

### Simulation-derived binding curves

**Figure 4:**
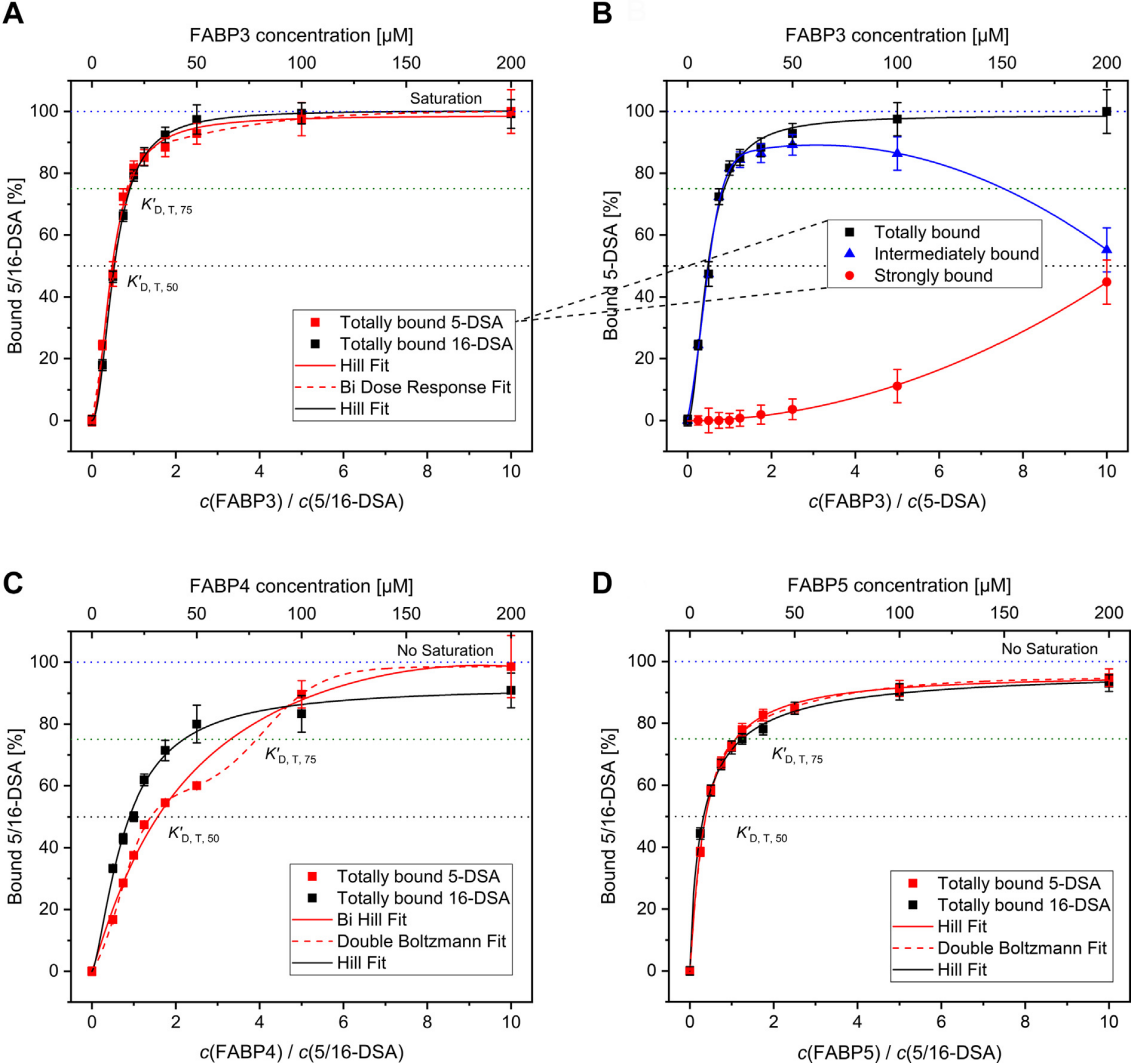
**Simulation-derived binding curves.** Curves for FABP3 (*A*), FABP4 (*C*) and FABP5 (*D*) with totally bound 5-DSA (*red*) and 16-DSA (*black*) are shown. For FABP3 with 5-DSA the curve is also shown separated into intermediately and strongly bound component (*B*). The curves were fitted using Hill and Bi Hill (*black* and *red solid lines*, respectively), Bi Dose-Response, and Double Boltzmann functions (*red dashed lines*, respectively).

The fractions (weights) Φ_x_ of the spectral components were extracted from the EPR simulations of the concentration series and were used to calculate the concentrations of all components with Eq (1). in Figure 3. This was performed for the intermediately and strongly bound components, as well as for their sum, called totally bound 5/16-DSA, to construct binding curves for all FABPs and spin probes. The binding curves for the FABPs with totally bound 5/16-DSA are shown in Figure 4, *A*, *C* , and *D*. Additionally, the curves of intermediately and strongly bound 5-DSA are shown separately in Figure 4*B* for FABP3. The curves for both spin probes and all FABPs can be found in Fig. S5. Different fit functions were applied to the curves for a better qualitative and quantitative analysis. For various binding curves two different fit functions could be applied, some of them show additional plateaus within their slope. However, the reliable occurrence of these binding plateaus is questionable due to the low number of data points in these concentration areas.

Generally, it is visible that higher protein concentrations lead to higher proportions of totally bound ligands. The curves always show a sharp increase up to a ratio of 1:1 between FABP and 5/16-DSA. Thereafter, the curve flattens out and reaches a plateau at approximately a twofold concentration excess of FABP, approaching the maximum of 100% bound molecules at a tenfold protein excess. However, complete saturation with 100% of bound molecules is only reached by FABP3 with 5-DSA. The curve slope is significantly different between the isoforms. The steepest increase can be found for FABP3, while FABP4 shows the lowest increase and FABP5 is intermediate between them. Interestingly, FABP4 shows a deviation in the curves of 5- and 16-DSA while they are almost similar for FABP3 and FABP5. The *K*_D, T_ values in Table 2 confirm these first observations.

**Table 2:** List of apparent and calculated *K*_D_ values [μM] for 100 μM of FABP3, FABP4 and FABP5 with 5/16-DSA

*K*_D_ and Δ*G*	FABP3		FABP4		FABP5	
Ligand	*5-DSA*	*16-DSA*	*5-DSA*	*16-DSA*	*5-DSA*	*16-DSA*
$K_{D,T,50}^{\prime }$ (fit)	9.6 ± 0.1	10.6 ± 0.1	31.6 ± 0.3	17.8 ± 0.2	6.6 ± 0.1	7.6 ± 0.1
$K_{D,T,75}^{\prime }$ (fit)	17.4 ± 0.2	18 ± 0.2	66 ± 0.7	43.6 ± 0.4	25 ± 0.3	22 ± 0.2
$K_{D,T,20\mu M}$	0.82 ± 0.65	1.08 ± 0.55	20.83 ± 0.8	10 ± 0.75	2.05 ± 0.5	2.14 ± 0.7
$K_{D,T,50\mu M}$	2.44 ± 1.87	0.81 ± 2.46	25.33 ± 1.0	8.5 ± 4.1	5.82 ± 1.14	5.87 ± 1.21
$K_{D,T,100\mu M}$	2.06 ± 2.68	0.48 ± 7.08	9.53 ± 0.53	16.7 ± 0.44	7.79 ± 0.37	8.9 ± 0.34
${\Delta G}_{F-T,100\mu M}$	−33.8 ± 3.4	−37.5 ± 5.4	−29.8 ± 1.1	−28.4 ± 0.9	−30.3 ± 0.8	−30.0 ± 0.8
${\Delta G}_{F-S,100\mu M}$	−18.4 ± 0.2	−13.1 ± 0.1	−20.3 ± 0.1	-	−20.8 ± 0.1	−19.4 ± 0.1
${\Delta G}_{F-I,100\mu M}$	−28.6 ± 0.9	−33.7 ± 2.5	−25.9 ± 0.4	−28.4 ± 0.9	−25.7 ± 0.2	−26.7 ± 0.3
${\Delta G}_{I-S,100\mu M}$	5.3 ± 0.3	10.6 ± 0.2	3.1 ± 0.2	-	2.6 ± 0.1	4.0 ± 0.1

Δ*G* values [kJ mol^−1^] were derived from the calculated *K*_D_ values for different transitions at 37 °C. Values of empty fields could not be calculate since the including component does not appear in the systems.

From the fit functions of the binding curves in Figure 4, apparent *K’*_D_ values were read out at 50% and 75% of the totally bound component. Furthermore, *K*_D_ values were also calculated by Eq (2). for 20, 50, and 100 μM FABPx. Finally, for 100 μM FABPx, molar Gibbs energies Δ*G* at 37 °C were calculated from *K*_A_ *via* Eq (4). for all possible transitions (see also Fig. 3). All results are shown in Table 2 and are analyzed below.

At this point, it is necessary to notify some assumptions for the EPR-derived binding parameters. Firstly, 5/16-DSA are conceived to have an EPR activity of 100% in our experiments, meaning that all molecules bear an active nitroxide group and no dimerization or further inactivation of the radicals has occurred. Secondly, micelle formation of 5/16-DSA in solution which would lead to peak broadening within the CW EPR spectra was neglected since the ligand concentration was held relatively low. This was checked *via* EPR reference measurements. The EPR simulations focused mostly on changes in the three spectral fractions (weight), mostly. However, additional changes in the parameters *g*, *A*, and τ_c_ cannot be excluded. In some cases, similar spectral shapes can be simulated by different parameter variations. For the calculation of equilibrium *K*_D_ values for all possible transitions we used identical and quite simple binding models of two interacting binding partners in total equilibrium (FABP and FA). Thereby, it was assumed that the ligand and macromolecules can be arbitrarily exchanged in the calculation. This could lead to a conflict with the consideration of intermediate binding of multiple molecules and possible cooperativity effects (see below). The attachment (intermediate binding) was validated as a separate binding process. Assuming that the fraction of totally bound 5/16-DSA includes strongly and intermediately bound molecules, the calculation could be partly uncertain since the underlying binding models, at least for intermediate binding could be optimized. More complex binding models could be required for a fully realistic representation of the binding equilibria. Regarding to the calculation of thermodynamic parameters *via* van’t Hoff equation, the calculation of *K*_D_ values includes the total protein concentration and the concentrations of free and bound ligands for one system and temperature. In this simple binding model, the system reaches full binding equilibrium at a constant temperature during the incubation and measurement time. Finally, the van’t Hoff equation is only completely valid for standard conditions since temperature- and aggregation-related changes of the protein functionality are not considered.

The order of *K*_D_ values for totally bound 5/16-DSA in Table 2 seems to be similar to that observed for rhodamine-PA in the MST experiments. For 100 μM of FABPx with 5-DSA the values are even very close to the corresponding MST results. The ${\Delta G}_{F-T,100\mu M}$ values are also in the same area as the Gibbs energies obtained from MST, confirming the exergonic nature of total binding. However, through EPR spectroscopy a more in-depth look into the thermodynamics of the additional transitions is possible. Direct strong binding (F-S) seems to be exergonic as well, but less favored with higher Δ*G* values, while the intermediate binding (F-I) is thermodynamically preferred. The sum of both binding transitions (F-T) shows consequently the highest negative Δ*G* values. Interestingly, the transition I-S is energetically located at the edge of exergonic and endergonic processes but is clearly endergonic at 37 °C. Accordingly, the transition from intermediate to strong binding is thermodynamically not favored at body temperature and should not occur spontaneously, whereas the inverted transition (S-I) is exergonic. This observation emphasizes the assumed role of intermediately bound 5/16-DSA potentially reflecting an activated transition state. The transition from the strongly to the intermediately bound state may well be described as an activation of the ligand for an easier release. Therefore, it is logical that such an activation should require a low energy barrier at the working temperature of the protein.

When comparing the *K*_D_ values obtained from the binding curves directly (Table 2) and the calculated values at different protein concentrations, strong deviations become visible. The graphic method appears to feature a relatively high margin of error, again due to the limited number of data points. While the apparent *K’*_D_ values at 50% of the bound component mark FABP5 as the protein with the highest affinity towards 5/16-DSA, at 75% of the bound component the affinity order becomes equal to that in the MST results. Accordingly, all directly read-out and calculated *K*_D_ values show a strong dependence on the total protein concentration. This observation is possible since binding affinities are thought to be statistic values which should depend on the size of an ensemble of binding partners.

The binding curves of FABP4 show lower levels of intermediately bound components with only small proportional changes below 100 μM (5-DSA) or 20 μM (16-DSA), respectively. Obviously, FABP4 has the lowest affinity towards 5/16-DSA if there is a deficit or a small excess of protein. The increase of the respective species of totally bound 5/16-DSA is significantly reduced for 5-DSA when compared to 16-DSA. This is in contrast to FABP3 and FABP5, where these curves are relatively similar. Besides an overall reduced affinity of FABP4 towards FAs, this suggests an even lower binding affinity of FABP4 towards 5-DSA than towards 16-DSA. At fivefold and tenfold excess of FABP4 this behavior is reversed since 5-DSA appears to be bound in higher proportions than 16-DSA. Generally, all FABPs bind comparatively more 5-DSA in total than 16-DSA at a tenfold protein concentration excess. However, when lower FABP concentrations are examined, this trend remains only for FABP5, but is reversed for FABP3. These observations are intrinsically mirrored in the calculated *K*_D_ values in Table 2. The difference in the binding affinity between 5- and 16-DSA could originate from the DOXYL group, disrupting (or promoting) interactions of amino acid residues with the alkyl chain nearby position C5 or C16. Probably, 16-DSA is generally less strongly and more intermediately bound than 5-DSA. This is reflected by the Δ*G* values for the transitions F-I and S-I for 5- and 16-DSA in comparison. Accordingly, the DOXYL group seems to interfere more significantly with the strong binding of the fatty acid when positioned at position C16 than at C5. Possibly, this could stem from interactions of the FABP with the alkyl chain near C16 being involved more heavily for the strongly bound state. This hypothesis would indicate that hydrophobic/dispersion interactions have a more important role in fatty acid binding than generally presumed.

The steep increase of the binding curves over a small concentration range indicates the cooperativity of binding. Since it is known that only one ligand is bound within the binding pocket of FABPs we assume that no direct cooperativity between strongly bound ligands can occur. However, cooperativity *e.g.*, by aggregate formation or intermediate binding of multiple ligands is still possible and seems to occur here with high probability. Aggregate formation could be responsible for the creation of new (intermediate) binding sites. Besides other models, the EPR binding curves shown in Figure 4 were also fitted using Hill equations. The analysis of the Hill fitting parameters shows that FABP3 and FABP4 binding curves exhibit Hill coefficients n >1 for 5- and 16-DSA, indicating positive cooperativity. The former description of the steep and high increase of the FABP3 binding curve already implied this conclusion. As an opposite, the Hill coefficient for FABP5/5-DSA is ≈1 and for FABP5/16-DSA <1, indicating no or even negative cooperativity in the case of FABP5 with 16-DSA. The FABP5 curves show a steep increase but faster flattening than in the case of FABP3 and FABP4. Cooperativities could occur between surface-attached and strongly bound or between various attached molecules. A positive cooperativity of intermediately bound ligands would be conceivable, but also a repulsive competition at the portal region which could lead to negative cooperativity. Furthermore, cooperativity with self-binding between FABPs (aggregation) could be also possible and will be discussed below. All these effects could lead to the observed concentration dependence of the *K*_D_ values. As a next step, a more in-depth analysis of the separated binding states and the differences between binding of 5- and 16-DSA can be performed for all three FABPs.

The binding curves in Figure 4*B* indicate, that intermediately bound fatty acids are increasingly ‘exchanged’ by strongly bound fatty acids above 50 μM of FABP3. In the case of FABP5, strongly bound fatty acids initially appear earlier, namely already at 25 μM. However, intermediately and strongly bound fatty acids both remain on a relatively constant level at high FABP5 excess and are not further exchanged. Therefore, the totally bound fraction is stabilized at 94.8% and 93% of 5- and 16-DSA, respectively. The visible differences within the 5/16-DSA spectra at low FABP5 concentrations (5–20 μM) and the steep curve rise of FABP5 indicate that the affinity of FABP5 towards 5/16-DSA could even exceed the affinity of FABP3 at the beginning. However, this is only valid in the range of very low FABP concentrations. The observation is attributed to the steep increase of intermediately bound component at low concentrations, before it decreases again and reaches a plateau, while the strongly bound component increases rapidly. This behavior of the intermediately bound component shows similarities to binding processes with negative cooperativity (as discussed before). Other binding-detection techniques such as MST are not able to resolve these differences at the same level of precision. Accordingly, the Δ*G* values of F-S and F-I for 100 μM FABP5 indicate that especially strong binding is preferred by FABP5 at higher concentrations while the thermodynamic preference of intermediate binding is slightly reduced compared to FABP3 and FABP4. These combined effects result in a faster saturation of FABP5 and an earlier plateau formation of the totally bound 5/16-DSA at high protein excess.

### Effects of medium choice, material source, and ligand competition

Inspired by questions in the review process of this paper, we also tested whether buffer choice, pH value, and protein source affect the results of our binding studies. For a full quantitative analysis of these effects on binding affinities, extensive studies with high amounts of proteins would be necessary, exceeding the actual scope of this project (see Outlook). Therefore, we focused on a few selected CW EPR measurements to obtain first, qualitative information on whether different buffer and pH values affect the spectra of 16-DSA in a mixture with the FABPs and we deduced whether there is an effect on the binding affinity, too. To this end, the sample FABPx/16-DSA 20/20 μM was used as a standard sample, measuring and comparing the CW EPR spectra in HEPES/NaCl at pH 7.5, pH 6 and pH 9, as well as in TRIS/NaCl at pH 7.5 for all FABPs. Alternative FABP3 with His-Tag and low glycerol content was also tested and showed a generally decreased binding affinity. We assume that attached glycerol molecules change the solvation and dynamics of the spin probes and thus impede 16-DSA from entering the binding pocket. For future studies, we recommend high caution when comparing binding studies of proteins with and without glycerol content. It should be noted that FABP4 and FABP5 were purchased from a different manufacturer for the test of pH and buffer, limiting the comparability with the main results. However, the new FABP5/16-DSA spectra show high similarity with the original spectra and the same pH/buffer dependence as FABP3 (see Fig. S6). FABP3 and FABP5 show higher binding affinities in HEPES pH 6 and a slightly higher one in TRIS pH 7.5 compared to HEPES pH 7.5. In HEPES pH 9 no changes in the spectrum appear. Spectral simulations for FABP3 and FABP5 reveal a 16 to 18% increase of intermediately bound component when lowering the pH from 7.5 to 6 and a 6 to 8% increase when changing the buffer from HEPES to TRIS, while no changes of the strongly bound component were simulated at the observed concentrations. The apparently higher binding affinities at lower pH values are consistent with literature results and could be connected with a higher proportion of protonated 16-DSA molecules when approaching the pKa value of 16-DSA/SA, affecting the binding affinity by the formation of new hydrogen bonds within the binding pocket (30). In contrast, the new FABP4 shows a strong deviation of the spectrum compared to the original data, with a significantly higher proportion of bound components (see Fig. S6*B*). In this case, we adjusted the concentrations of the samples by comparing the new CW EPR spectra with the original ones and we selected the nominal concentration ratio of 10/20 μM for buffer and pH comparison. FABP4 seems to have the highest affinity in HEPES pH 7.5 and TRIS pH 7.5, while it is reduced at pH 6, pH 9, and in pure water. We substantiate the deviations with contaminations or stabilizing additives from the manufacturing process of the new FABP4. To obtain clearness about this circumstance we performed a native gel PAGE and an SDS-PAGE (see Fig. S17), both gels exhibit relatively strong bands at 60 to 70 kDa. The SDS-PAGE confirms that these special bands do not appear due to aggregates but rather fit well with the size of monomeric serum albumin (66 kDa). Since also the CW EPR spectra show similarities with 16-DSA bound to serum albumin we cautiously suspect albumin impurities (or rather major components) are contained in the purchased product (30). Therefore, (only) the results of the FABP4 buffer and pH dependence should be treated with caution, while the native gel that we performed can be discussed independent of that. The observation also emphasizes the usefulness and sensitivity of CW EPR spectroscopy compared to other binding methods and demonstrates that spin probing with 16-DSA can be indirectly utilized for protein analytics. In summary, we demonstrated with our additional analysis that buffer and pH conditions can have effects on the results of CW EPR spectroscopic studies and binding studies in general. This important information should be kept in mind for future studies. Furthermore, we found a high dependence on manufacturing conditions for FABP4, which could be disturbing for the comparison of results from binding studies in general. As one may expect, it is therefore highly recommended to use material from consistent manufacturing conditions and standardized expression and purification routes for such kind of sensitive studies, including own purity tests if necessary.

In a second addition during revision, we were curious about the question of whether our model ligands that bind with medium affinity to the FABP binding pocket will be displaced when we add a native ligand with higher affinity and whether we could detect this displacement with our CW EPR setup. The native ligand DHA was chosen for this displacement analysis since it is well-known to bind with high affinity to FABP5 (26). DHA was pre-dissolved in ethanol, diluted into buffer, and mixed with FABP5 and 16-DSA. Reference measurements of FABP5 and 16-DSA with ethanol of the same concentration below 0.1 vol-% demonstrated no effects of the residual ethanol on the spectra. Samples with FABP5/16-DSA/DHA ratios 20/20/0, 20/20/5, 20/20/10, 20/20/20, 20/20/50, and 20/20/100 were prepared with 5 min incubation time in between 16-DSA and DHA addition. The CW EPR measurements showed a small decrease of bound 16-DSA from 72% to 55% with rising DHA content, but to our surprise, no complete displacement of 16-DSA occurred (see Fig. S7). We assume that the well-known high binding affinity of DHA refers to the state of strong binding within the pocket. Intermediate binding, which is dominant here, does not seem to mirror the affinities of 16-DSA and DHA, otherwise, complete detachment and only free 16-DSA would be detected, *e.g.*, at the ratio 20/20/100. Instead, a dynamic exchange of ligands seems to occur, as we consistently observe it with albumins from different species. However, this part opens the way to other future competitive binding assays *via* CW EPR spectroscopy, *e.g.*, with pharmaceuticals that bind to FABPs.

To sum up the last section, the common model for the binding mechanism of FABPs could be refined by the detection of two differently bound components. Interestingly the interplay between intermediately and strongly bound fatty acids differs between FABP3, FABP4, and FABP5 in different concentration ranges. An explanation of the different affinities towards both binding states between the three FABPs by single amino acids is not possible so far, due to the low sequence identities. Considering the fact that labeled stearic acid and palmitic acid both show the same affinity order, the FABPs seem to have an encoded binding selectivity for long-chain fatty acids. If total binding would be specialized through sequence diversification, it would be also assumable that intermediate binding could be specialized as well. However, the observed differences in ligand attachment could also occur as a side effect without special purpose. The following section extends these new findings and we want to shed more light on the nature of intermediately and strong binding state by probing the binding from a temperature-dependent viewpoint.

### Temperature-dependent changes in the binding of fatty acids

#### Temperature-dependent CW EPR spectra, simulations, and binding curves

**Figure 5:**
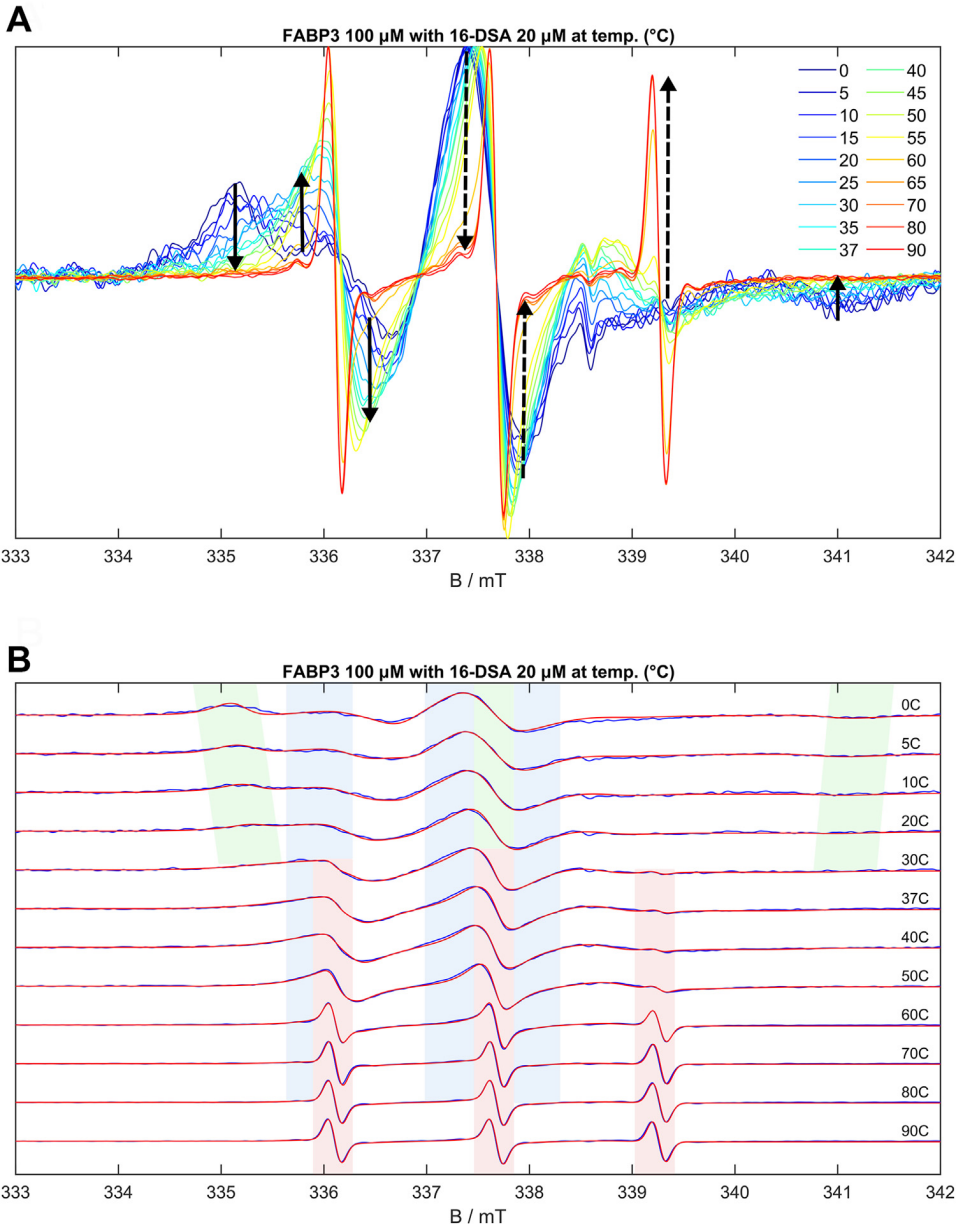
**Temperature series of 100 μM FABP3 with 20 μM 16-DSA.***A*, CW EPR spectra series normalized on the maximum intensity, *solid arrows* show low-temperature transitions, *dashed arrows* show high-temperature transitions. *B*, CW EPR spectra (*blue lines*) with EPR simulations (*red lines*) for selected temperatures.

While the previously shown CW EPR spectra were measured at 37 °C, the FABPx-5/16-DSA systems were also analyzed in a temperature range of 0 to 90 °C. Studies of the binding behavior and ligand dynamics beyond physiological conditions might reveal important information about the thermodynamics of the binding process and the stability of the proteins. The binding of fatty acids undergoes strong temperature-dependent changes, as *e.g.*, shown by the spectra and simulation series of 20 μM 16-DSA with 100 μM FABP3 in Figure 5. Further temperature series of 5- and 16-DSA with all three FABPs can be found in Figs. S8–S10.

**Figure 6:**
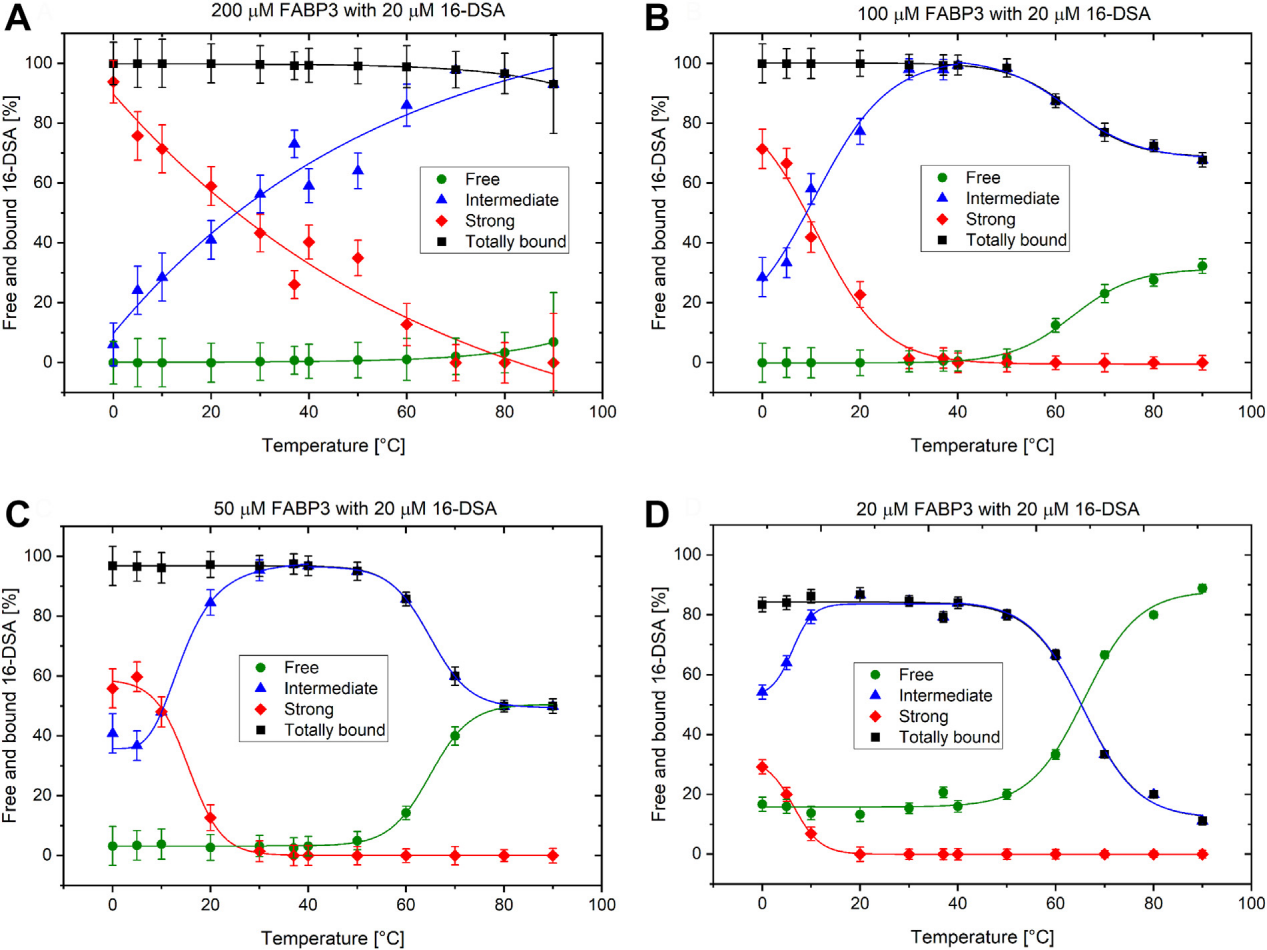
**Temperature-dependent binding curves of FABP3 with 16-DSA.** Curves for the concentration ratios 200/20 (*A*), 100/20 (*B*), 50/20 (*C*) and 20/20 (*D*) µM are shown. The fit functions (*solid lines*) are meant to guide the eye in this figure.

The simulations in Figure 5 and the derived temperature-dependent binding curves in Figure 6 and Figs. S13–S15 allow a full analysis of the ligand binding situation from 0 to 90 °C. Below 0 °C the solutions are frozen and the ligands are bound by the proteins. The radicals are mostly immobilized and powder-type spectra with relatively low signal-to-noise ratios can be found. Examples of FABP3 and FABP5 with bound ligands at very low temperatures are given in Fig. S11, *A* and *B*. At 0 to 5 °C the solution is thawed but highly immobilized molecules dominate in an extent depending on the protein/ligand ratio used. One should be cautious when assigning the binding state of these fatty acids, though. Highly immobilized DSA molecules may dominate the spectra at these temperatures even when there were only intermediately bound fatty acids. The association with the FABP proteins (which at 37 °C may be intermediately immobilized) in a low-temperature solution slows down their overall rotational motion. Visually, even for low FABP/ligand ratios such as 20/20 μM highly immobilized ligands are present in low proportions at these temperatures. In simulations, they can be separated from intermediately bound components *via A*_zz_ and τ_c_ (described in detail below). Since these immobilized components are also present in significant amounts at 20 °C and above, one may safely assume that at least parts of the highly immobilized spin probes certainly correspond to strongly bound fatty acids. The probably most interesting transition found in the temperature-dependent binding curves occurs when increasing the temperature from 0 to 37 °C (marked by solid arrows in Fig. 5*A*). A gradual exchange of strongly bound by intermediately bound (not freely rotating) 5/16-DSA can be seen in this range. Finally, the intermediately bound component becomes the dominant one, the temperature of this change depends on the FABP concentration. Around 37 °C the strongly bound components often reach a minimum, except for very high FABP concentrations, while the proportions of intermediately bound 5/16-DSA are maximized. Beyond body temperature, in a range of 40 to 90 °C, two different temperature behaviors for the FABPs and concentration ratios were observed. (a) The 5/16-DSA can be gradually released by the protein, starting at 50 to 60 °C, a temperature area that is connected with characteristic changes of the spectral shape within various temperature series. This release does not occur completely and is indicated by a continuous increase of free 5/16-DSA at the expense of the bound component until the proportion of bound 5/16-DSA reaches a minimum at 90 °C (marked by dashed arrows in Fig. 5*A*). The FABP3/16-DSA systems in Figures 5 and 6 show such behavior as well as FABP4 with 5/16-DSA (Fig. S9). (b) Alternatively, the ligand is kept bound inside the probably denaturing protein and the radical is destroyed at high temperatures, at least the EPR-active nitroxide moiety, leading to a signal loss. This was found *e.g.*, for 200 μM of FABP3 with 5-DSA (Fig. S11*C*) and for 100 to 200 μM of FABP5 with 5-DSA (Fig. S10*B*). The examples with FABP5 show a permanent exchange between free and bound 5/16-DSA before the stepwise signal loss occurs (Fig. S11*D*). This effect leads to deviating peak intensity maxima at 50 to 60 °C and not at 80 to 90 °C as it is often found for FABP3. Generally, at lower FABP concentrations and for 100 μM FABP4 with 16-DSA, mainly changes in the ligand dynamics can be seen in the spectra.

### Analysis and discussion of the temperature-dependent binding process

As demonstrated before, the intermediately bound component reaches its maximum contribution in the temperature range of 35 to 45 °C for most of the analyzed systems. This range corresponds closely to the physiological temperature of the human organism. The observed intermediately bound component, interpreted above as ligands that are attached to the protein surface or to the portal region, could also represent a thermodynamic transition state of the ligand as part of the binding mechanism. This transition state could occur as protein surface adsorption of the fatty acids prior to entering the binding cavity, as it was suggested by Friedmann *et al.* (39). From a kinetic viewpoint, the existence of a transition state has been also previously suggested by Richieri *et al.* (40), after performing fluorescence experiments. There, a protein conformational change has been proposed as the basis of the transition state. The results obtained in this work could finally connect and experimentally prove the previous theories. Human FABPs should reach their highest activity at body temperature, as reflected by the maximum of the intermediately bound component. While the strongly bound state could be optimum for the successful transport of hydrophobic ligands, the intermediately bound state should thermodynamically be more favorable during binding and release of the ligands. This has already been shown with the Δ*G* values at 37 °C in Table 2. Facilitated binding, activation, and release require a low energy barrier, while permanent strong binding would be inappropriate for the transport function of the FABPs. As it was done for the binding curves at 37 °C, Δ*G* could now be also calculated for all other temperatures. Moreover, van’t Hoff plots would enable the analysis of thermodynamic binding parameters for all equilibrium transitions. This approach was previously used for core-shell polymers by Reichenwallner *et al.* (41) and could be adapted now to analyze the thermodynamics of fatty acid attachment and binding by FABPs on a fundamental level. This would expand the already existing thermodynamic analyses of FABPs by Richieri *et al.* (42, 43). However, such a detailed thermodynamic analysis is beyond the scope of this initial study, with which we aim at a clearer illustration of the binding processes on a molecular level, and will be reported in a subsequent study.

Since the ranges of low (0–10 °C) and high (60–90 °C) temperatures are not as relevant for the human organism, they will not be discussed in much detail here. However, few species are known with slightly or strongly deviating body temperatures. The FABPs of the icefish, for example, were analyzed at the freezing point of water in former studies, where no significant change in the *K*_D_ value was found (44). Even though human FABPs have adapted and optimized their binding characteristics to the body temperature of 37 °C, this may not be the case for other organisms in different environments. It is therefore worthwhile to study the behavior of FABPs at extreme temperatures, too, especially considering that the proteins may have originated from an ancestral gene that possibly evolved under different environmental conditions (1). Apart from that, the basic thermodynamic properties of the proteins can only be seen by the analysis of a wide temperature range, including extreme conditions. It is conceivable that the FABPs start to denature above body temperature. Thereby, the association between FABP and fatty acid is disturbed, finally resulting in the release or detachment of the fatty acid. Subsequent EPR measurements at 37 °C after ending the temperature series showed that a small proportion of 5-DSA survived the high temperatures and was able to bind to the residual protein again, or that it remained strongly bound even though the signal intensity was decreased dramatically (see Fig. S12). This occurred, for example, in the case of FABP4. Since the binding seems to be partly reversible, one may assume that the FABPs have a relatively high functional stability against extreme temperatures. The irreversible signal loss that occurs for some systems at high temperatures (‘trap and destruction’) may be explained by a different denaturing process, or a reaction of the radical, leading to an irreversible deactivation of the ligand and/or destruction of the FABP. In this case, no certain conclusions can be drawn for the temperature stability of the FABP by the used methods. In summary, the FABP isoforms show different temperature (binding) behavior and thermodynamic properties that may be related to their functionality. Another important question is whether FABPs also bind to each other together with or instead of fatty acids, a process that is known as self-aggregation. For a comparison with the binding studies, the same parameter dependencies were analyzed in the aggregation studies.

### Self-aggregation of the FABPs

First indications for self-aggregation of the studied FABPs were found in the MST measurements, by analyzing the shape of the MST traces in Fig. S2, *B*, *D*, and *F*. Comparing the traces, they show the highest deviation from a perfectly smooth shape for FABP3 in buffer, followed by FABP4 in water, and finally FABP5 in buffer. For the latter, almost perfectly smooth traces were measured. The possible formation of micelles by rhodamine-PA can be neglected due to the low concentration of 80 nM used. Thus, the proteins have to be responsible for this observation. In this context, the influence of the solvent and the salt condition must be discussed in brief. FABP4 showed a strong turbidity formation and precipitation at protein concentrations between 20 μM and 200 μM in HEPES/NaCl buffer at pH 7.5, which was not observed for the other FABPs. The used buffer or the salt concentration might be inappropriate for this FABP, however, this is not analyzed in detail here. To avoid this problem, FABP4 was handled in water. The MST traces of FABP3 and FABP4 also appear to be less smooth with increasing measurement time. This effect could occur due to the irradiation of the sample by the IR laser, which heats the solution in the capillary and thus could affect the protein itself.

#### Isoform-dependent particle radius distributions

**Figure 7:**
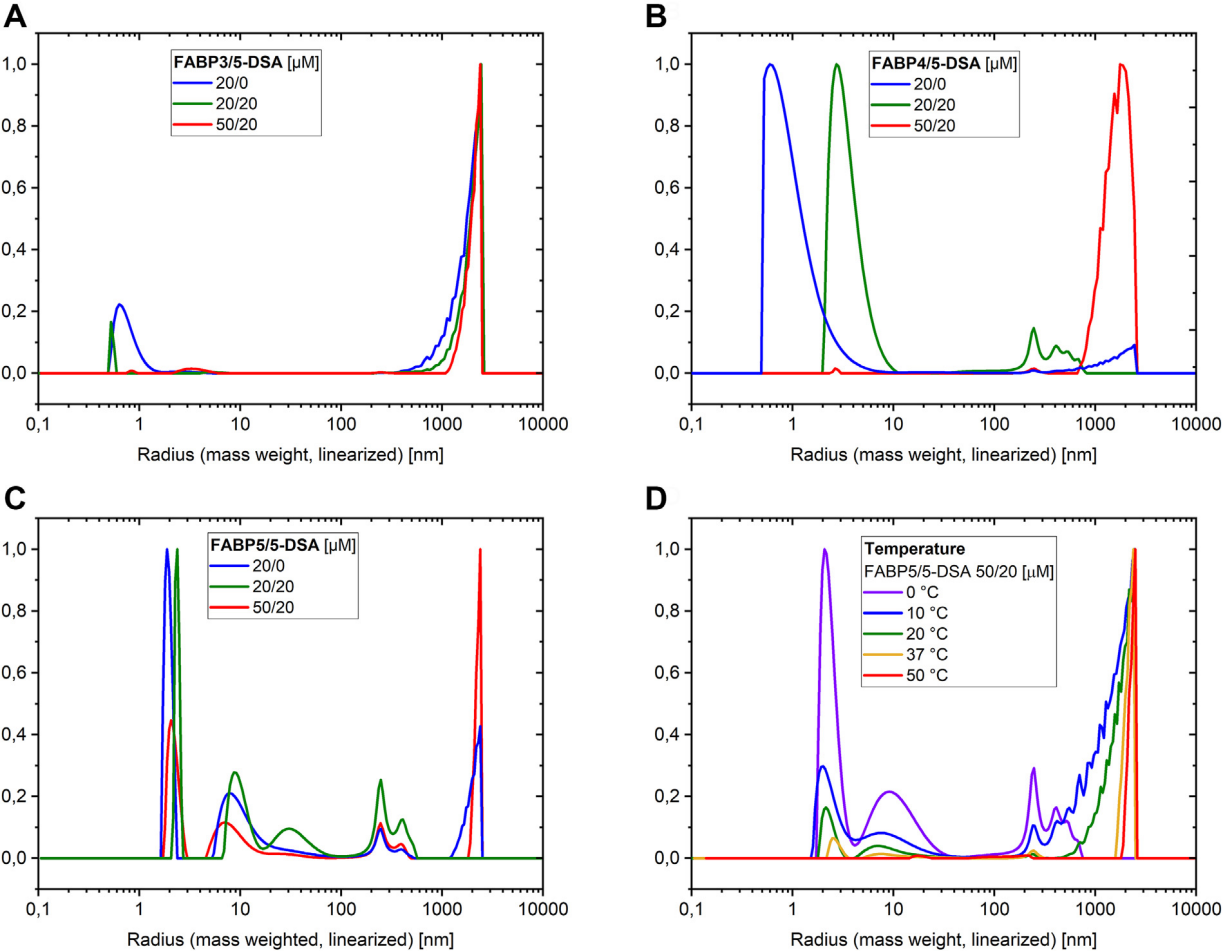
**Particle radius distributions for FABPx/5-DSA mixtures.** Distributions for different concentration ratios were measured *via* DLS at 37 °C (*A*–*C*). Temperature-dependent particle radius distributions were measured for the sample FABP5/5-DSA 50/20 μM (*D*).

For a closer look into possible aggregation effects, the hydrodynamic radii of FABP particles in solution were measured by DLS. The particle radius distributions in Figure 7 indicate the formation of FABP dimers and large aggregates in buffer (FABP3, FABP5) and water (FABP4) at 37 °C. Note that at the low 5-DSA concentration used here, no micelle formation or other structure detectable in the solution arises from only the stearic acid derivative. Hence, all changes observed always stem from either the protein or protein and 5-DSA structure formation.

While all peaks above 10 nm are assumed to be medium-sized and large protein aggregates, the recurring radius peaks at 1 to 2 nm can be interpreted as monomeric protein. Obviously, their weighting in the distribution decreases with rising concentration of the FABP due to aggregation. The extent of the aggregation depends on the FABP isoform and the concentration, as well as the concentration of added ligands and the temperature (see Fig. 7*D*). FABP3 in buffer shows the strongest aggregation, while FABP4 in water shows a much lower degree of aggregation but a higher polydispersity. This observation confirms that the aggregation does not only occur due to buffer conditions but could be decreased in a less polar environment. In contrast to the other FABPs, FABP5 is well soluble in buffer and shows less aggregation with lower polydispersities, especially at 50 μM. An increase in the protein concentration results in a higher amount and larger size of the aggregates, while the proportion of small particles is reduced. Interestingly, the addition of 20 μM 5-DSA seems to reduce the aggregate radii, but increases the radii of small particles. This behavior was observed for FABP4 and FABP5, but not for FABP3. The addition of 5-DSA shifts the radius of monomeric FABP, possibly arising from the attachment of 5-DSA molecules to the surface of the protein. Along with water molecules, the added 5-DSA could generate a large solvation shell. This could also explain the relatively high polydispersity of the particle radii. However, another interpretation of the radius shift could be the promoted formation of dimers, since the recurring radii of 2 to 4 nm are in the size range for such protein dimers. In this case, the binding of 5-DSA would support the aggregation of the FABPs, perhaps through a form of physical bridging effect between FABP molecules. As a consequence, ligand binding and self-aggregation could not be competitive, but rather synergistic. This hypothesis should be further investigated in future studies. The particle radius distributions of FABP4 and FABP5 reveal that the addition of 5-DSA to a constant concentration of FABP promotes the formation of medium-sized aggregates (250–500 nm), but reduces the amount of large aggregates above 1000 nm. With increasing protein concentration this effect is reversed again. The sharp peak at 250 nm appears in all FABP5 and in some of the FABP4 distributions. Its appearance without 5-DSA (FABP5, 20/0) as well as in ultrapure water as a medium (FABP4, 20/0) indicates a recurring aggregate structure of all FABPs and does not speak for a measurement artifact, the buffer, or added 5-DSA. In the case of FABP3, the larger aggregates are generally dominant, even for low protein concentration. Theoretically, in sample 20/20 the highest ratio of ligand-loaded to unloaded proteins should occur, assuming the standard picture of only one bound ligand per protein. This could imply that ligand-loaded proteins have a higher tendency to organize themselves into medium-sized aggregates, whereas unloaded proteins tend to remain solitary or to form larger aggregates. Surface-attached fatty acids could also promote or disturb the aggregation. Based on this observation, one may hypothesize the existence of a hypothetical, specialized binding mechanism, involving ligand-FABP and FABP-FABP interactions. However, there is no possibility to derive more information about this potential interaction mechanism beyond this correlation with the methods used in this work. Although the formation of dimers by FABPs has been observed frequently (45), self-aggregation to this extent has only been reported for pig-FABP3, by Fournier *et al.* (35, 46). In these studies, at least three higher molecular weight aggregated species of FABP3 were found and associated with the concentration-dependent binding of ligands. This coincides with the results obtained in this work. However, competition between ligand-FABP and FABP-FABP binding was postulated (35), rather than a positive influence of aggregation on the ligand binding affinity. Further studies should be done to elucidate these contradicting observations. Details about the structure(s) of the aggregates remain unclear. For example, small beta-barrel domains (SBBs) are able to form highly ordered aggregates by partially opening their barrel structures (47), suggesting the possibility of a similar mechanism for the FABPs. However, FABPs have twice the number of β-strands, probably making them structurally less flexible.

#### The relation of protein aggregation and ligand binding

After observing self-aggregation by FABPs and finding that bound ligands could influence this effect, we intended to answer the inverse question, of whether FABP aggregates also bind the ligands differently than FABP monomers. Hence, we compared the DLS results with new CW EPR measurements. In contrast to former measurements, this time identical ligand-to-protein ratios but alternating total protein concentrations were used, to ensure different aggregation states but identical concentration ratios of the binding partners. CW EPR measurements of FABPx/16-DSA ratios 100/100, 50/50, 20/20 (standard), and 10/10 μM/μM were performed and compared with each other, normalizing the spectra on the same center field peak intensity (see Fig. S16). For FABP3 and FABP5 spectral simulations were done by adjusting the component proportions. The spectra of all three FABPs show strong increases in bound components with increasing total concentration and (derived from the DLS data) an increasing proportion of oligomers. The intermediately bound proportion of FABP3 increases from 67.1% (10/10) to 72.6% (20/20), 85.9% (50/50) until 90.3% (100/100). For 100/100 additionally, a strongly bound component was simulated with 2.8%. FABP5 shows a similar trend of intermediately bound 16-DSA: 61.5% (10/10) – 72.2% (20/20) – 80.8% (50/50) – 81% (and 9.5% strongly bound) (100/100). These results indicate that oligomeric FABPs which likely appear at higher protein concentrations as DLS data have shown, bind 16-DSA with higher affinity. The amount of bound component is proportionally increased with higher total concentration, but no changes in the dynamics of the bound component, and thereby the binding mode could be found. Therefore, the spectral changes do not just represent more strongly immobilized ligands through aggregate formation (which would not argue for binding-aggregation dependence) but rather show increased proportions of the same bound component, which argues for an increased binding affinity instead. It is likely that protein clusters would be able to enclose several ligands, leading to local protein-rich and ligand-rich microenvironments, *for example*, as a form of irregular FABP clusters with locally higher binding affinities towards fatty acids. These results corroborate our suspicion that ligand binding and aggregation are synergistic effects.

We intended to further analyze the possible relation between ligand binding and FABP oligomerization with a second method, choosing native gel analysis with new FABP4 and 16-DSA at different 16-DSA loadings. By running a native gel, the aggregates of FABP4 should become visible. As a reference without aggregates, we performed an SDS-PAGE which we used to check the purity of the new FABP4 at the same time. Both gels were loaded with FABP4 and 16-DSA in the same concentration ratios 20/0, 20/10, 20/20, 20/50, 20/100, 50/0, 50/20 and 50/50 μM, together with a molecular size marker (M). The native gel in Fig. S17*A* shows no FABP monomers (expected at 14–15 kDa), but oligomers at approximately 45 kDa which we identify as trimers of FABP4, as well as possible further oligomers at 60 to 80 kDa and higher aggregates above 100 kDa that probably stem from *Escherichia coli* bacteria proteins during the recombinant expression. In the SDS-PAGE in Fig. S17*C* we could identify the monomeric FABP4 at 14 to 15 kDa, but we also found again a strong band at 60 to 70 kDa, showing that this band cannot be contributed to FABP4 oligomers. In accordance with our discussion above, this band seems to be contaminated with another protein, possibly Serum Albumin (66 kDa). However, the oligomer band in the native gel strongly increases with increasing protein concentration and also seems to increase slightly with a higher ligand loading, at least from 0 to 20 μM 16-DSA. This result corroborates our observations from DLS and CW EPR spectroscopy, showing that ligand binding and oligomerization are synergistic. Furthermore, dilution of the FABP from 50 to 20 μM decreases the proportion of oligomers, as found in DLS, with the lowest oligomerization appearing at a loading ratio of 20/0.

#### Temperature-dependent particle radius distributions

The aggregation processes were also analyzed in dependence of temperature, in a range of 0 to 90 °C. Typical DLS radius distributions for one FABP5 sample are shown in Figure 7*D*. It is observed that monomeric or dimeric FABP molecules dominate below 20 °C, while large aggregates increase with rising temperature and finally become dominant. Above 50 °C only extremely large aggregates in the micrometer range, probable precipitates, are visible. This trend is found for all FABPs. However, for some FABP/ligand systems, a possible stabilization of smaller FABP particles is found at around 37 °C. This can be seen for 20 μM FABP5 with 20 μM 5-DSA in Fig. S18, but not for 50 μM FABP5 in Figure 7*D*. It is also found for FABP4, which is not included here. Accordingly, the effect seems to depend strongly on the ratio of FABP and 5-DSA, which in turn suggests a mutual influence between ligand binding/attachment and self-aggregation around body temperature. Furthermore, a slight difference is found between measurements at 37 °C during the heating process and single measurements directly at 37 °C. Therefore, both parameters, temperature and concentration ratio, affect the aggregation behavior of the FABPs in a complex manner.

It is not yet known which structural changes FABPs undergo at temperatures high above body temperature and how temperature stable they are in general. It is likely that some form of denaturation takes place. However, FABP5 and FABP4 samples show recurring peaks of small radii at the last measurements at 37 °C after heating up to 90 °C before. This indicates an incomplete or partially reversible aggregation. The fact that FABP3 does not show this behavior and the generally earlier aggregation at lower temperatures indicates a lower temperature stability of FABP3 compared to the other two FABPs.

### General discussion of FABP structure, dynamics, and polarity

**Figure 8:**
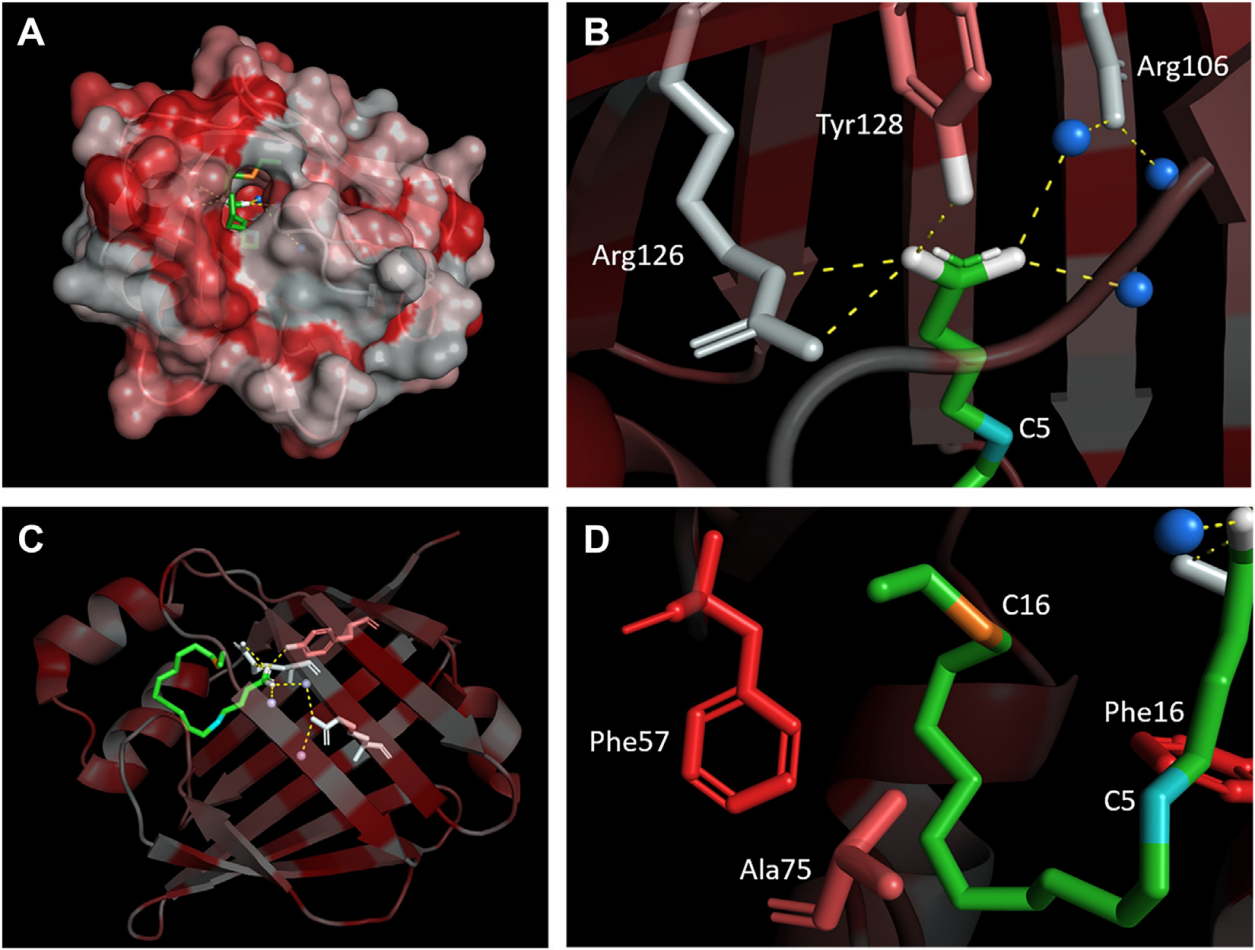
**Crystal structure of FABP3 in complex with stearic acid (*green*)** (48)**, colored by the hydropathy indices of the amino acids (*white* = hydrophilic, *red* = hydrophobic).***A*, surface and patch structure of FABP3 with a view into the binding pocket. *B*, view on the headgroup (*white*), the hydrogen bonds (*dashed yellow lines*) towards adjacent polar amino acid residues and water molecules (*blue spheres*) and the theoretical C5 position of 5-DSA from inside of the binding pocket. *C*, conformation of complexed stearic acid as seen from outside of the FABP. *D*, view on the alkyl chain of the stearic acid with the theoretical C16 position of 16-DSA and adjacent non-polar amino acid residues.

Beyond the binding and aggregation studies presented so far, further biophysical properties of the FABPs and their bound ligands can be analyzed with our employed methods. Especially the presented EPR simulations provide more in-depth information about the molecular dynamics and environments of the ligands. Therefore, a more holistic analysis starts with the dynamics of the bound ligand in comparison to intermediately attached and free ligands, then continues with the ligand’s environment inside and outside of the pocket and ends with a look at the surface of the protein. Figure 8 shows the crystal structure of FABP3 in complex with stearic acid from inside and outside of the binding pocket (holo-conformation), it serves as the reference point for the following discussions (48). All amino acid residues are colored by hydropathy indices using the scale of Kyte and Doolittle (49). It should be mentioned that FABP5 exhibits a disulfide bond between Cys120 and Cys127 within the binding pocket. Theoretically, it could react with the radical and reduce the available space within the pocket (50). However, no verifiable effect of the disulfide bond on the CW EPR spectra could be found in our studies. Since no systematic trends have been found for *g*_iso_ between different FABPs, concentrations, and temperatures, only the changes in *a*_iso_ or *A’*_zz_ and τ_c,iso_ will be discussed in more detail below. The tensor value *A’*_zz_ was evaluated since changes of it contribute to the isotropic *a*_iso_ value more significantly than changes in the other tensor values. In the discussion below *A’*_zz_ and *a*_iso_ are used equivalently.

#### Protein/ligand dynamics

The rotational correlation times of the radicals increase from free *via* intermediately to strongly bound 5/16-DSA (see Table 3). Stronger interactions with the FABP result in slower dynamics. Interestingly, intermediately and strongly bound 16-DSA show larger τ_c_ values and thus lower rotational dynamics than 5-DSA. Since 16-DSA has the more polar DOXYL moiety attached at the C16 position and 5-DSA at the C5-position, this difference in dynamics could be due tointeractions such as van-der-Waals-interactions between hydrophobic amino acid residues and the alkyl chain of the fatty acid somewhere between the carboxylic acid headgroup and the C16-position. Alternatively, the DOXYL group could prevent tighter association of the fatty acid for conformational or steric reasons when attached at C5.

**Table 3:** Simulated values for τ_c_ and *a*_iso_ for 5/16-DSA with all FABPs and components (comp.) at 37 °C

Simulated parameters		FABP3		FABP4		FABP5	
Parameter	Comp.	5-DSA	16-DSA	5-DSA	16-DSA	5-DSA	16-DSA
*a*_iso_ [MHz]	(1)	44.1	44.1	44.1	44.1	44.1	44.1
	(2)	42.9	41.6–42.6	46.9	44.7–48.0	44.9–46.9	37–42.3
	(3)	41.3	38.7	43.0	36.7	42.3–43.7	37
τ_c,iso_ [ns]	(1)	0.1	0.04–0.1	0.06–0.08	0.05–0.13	0.09–0.1	0.05–0.07
	(2)	4.8–4.9	5.2–8.4	4.8	4.0–5.8	4.8	5.7
	(3)	17.9–19	19.1–22.2	17.9	22.2	17.9	22.2

For a better legibility error values are not depicted here (see Experimental procedure for general percentage errors of the parameter values below).

As can be expected, with increasing temperature, the freely tumbling 5/16-DSA molecules become more dynamic and show a faster rotational motion due to higher kinetic energies (Fig. S19*A*). In the EPR simulations of the temperature series, this appears as a decreasing rotational correlation time of the free 5/16-DSA and increasing peak intensities.

Increasing rotational dynamics were also found for intermediately bound 5/16-DSA (Fig. S19*A*). The increased dynamics at higher temperatures may explain the stepwise detachment of intermediately bound, potentially surface-adsorbed, fatty acid molecules. Since the strongly bound component only rarely occurs at higher temperatures in general, no statement can be made whether the rotational motion of bound fatty acids in the binding pocket is increased before a complete detachment of the strongly bound fatty acids occurs. However, it is conceivable that also molecules bound inside the binding pocket become more dynamic, potentially leading to the release of the molecule from the binding pocket. Thus, one may describe this process as the transfer of a critical ‘activation’ energy for ligand release into the system to overcome van-der-Waals-interactions and hydrogen bonds. Especially the intermediately bound 5/16-DSA seems to be sensitive to temperature increases, it is more loosely bound and can be detached more easily (from the surface) than strongly bound 5/16-DSA from the inner space of the β-barrel.

It is worth noting that a stronger increase in the rotational correlation time of the intermediately bound component leads to the formation of two peaks beyond the low-field peak in simulations. Therefore, it could be possible that a fourth component with rotational dynamics between intermediately and strongly bound occurs at some temperatures which may not adequately be dealt with in the simulations. Furthermore, an alternative interpretation of the intermediately bound component would be also conceivable. Apart from the attachment of fatty acids on the protein surface, the component could theoretically represent a different conformation of the FABP, too. Previous simulations of apo- and holo-forms of FABP3 showed that the protein can adopt two conformations, a wide-opened or a closed conformation, leading to small deviations in the crystal structure (19, 48). In case of no bound ligand, the portal region is opening to the external solvent by hydrogen-bond cleavage and α-helix movement. When stearic acid is bound within the binding pocket, the portal closes and the internal solvent molecules are rearranged. This closed conformation is conceived to be stabilized *via* van-der-Waals-type protein-ligand interactions. Furthermore, also partially open conformations were found in these simulations when long-chain fatty acids filled the binding pocket but hindered the portal from closing completely (48). In the studies of Armstrong *et al.* (50), small changes of the portal conformation by bound ligands were interpreted as activation of the FABPs for translocation processes. Please note that no crystal structures of FABPs complexed with 5/16-DSA exist in literature so far. But assuming a similar conformation of our model ligands (see Ligand Conformation), the binding of 5/16-DSA could have a similar effect on the dynamic conformation of the protein which could influence protein aggregation and cooperative binding or attachment.

In this scenario, an intermediately bound ligand could appear as a (partially bound) transition state at the open portal region of the FABP. This kind of transition state would be also in accordance with the transition state suggested in the literature (40). However, this interpretation seems to be less likely considering the significant difference of τ_c_ between the I and S states, the fact that both appear simultaneously at various protein concentrations and temperatures and the different environmental polarities (described below). Finally, the aggregation state of the protein also influences the combined dynamics of the protein and ligand. Thus, the formation of slowly rotating aggregates should additionally decrease the rotational mobility at high protein concentrations and could contribute to the strongly bound state. However, the strongly bound component cannot be completely contributed to aggregation, since the temperature transitions ‘S-I’ in CW EPR and ‘monomers-aggregates’ in DLS are reversed, indicating a higher plausibility of the previous interpretation of the strongly bound 5/16-DSA. Furthermore, the dynamics of the strongly bound component do not seem to change with stronger aggregation.

#### Ligand conformation

**Figure 9:**
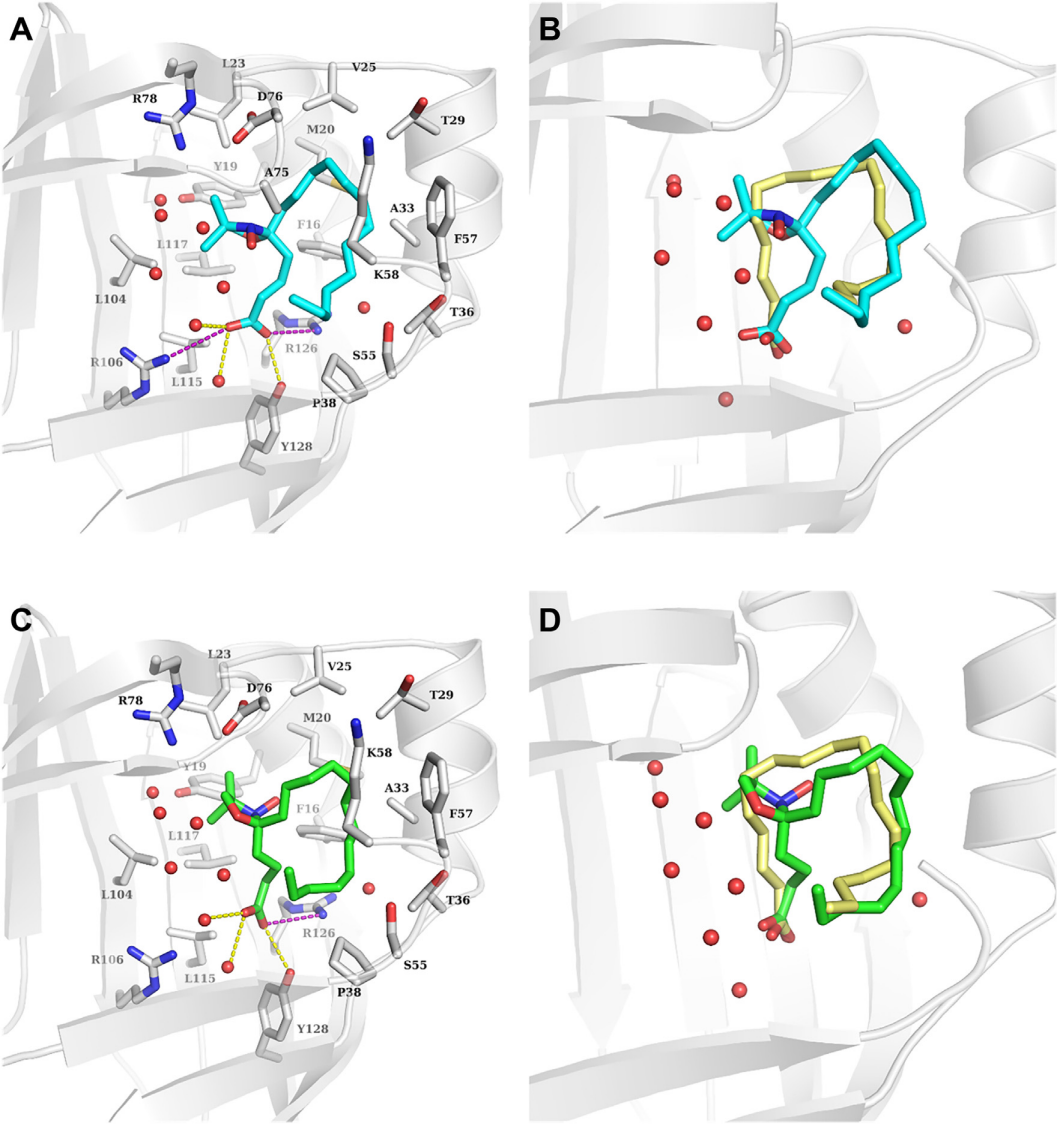
**Predicted binding modes of R- and S-5-DSA in the binding cavity of FABP3 (PDB: ID****4WBK****).***A*, docking pose of R-5-DSA (*cyan sticks*); (*B*) overlaid with the experimentally determined binding mode of stearate (*yellow sticks*); (*C*) docking pose of S-5-DSA (*green sticks*); (*D*) overlaid with the experimentally determined binding mode of stearate (*yellow sticks*). Relevant amino acids in the binding cavity are shown as white sticks and water molecules as *red spheres*. H-bond interactions are depicted as *yellow dashed lines* and salt bridges as *purple dashed lines*.

Whenever we perform binding studies with stearic acid derivatives the question appears whether strongly bound 5/16-DSA can be compared with native stearic acid with regard to its conformation when it is bound to the protein, in this case within the FABPs. Crystallographic studies have previously shown that long-chain fatty acids are accommodated in a U-shaped conformation into the binding cavity of FABP3, -4 and -5. The carboxylate group interacts with the side chains of the conserved Asp and Tyr residues as well as with two ordered water molecules. One side of the U-shaped fatty acid is lined by hydrophobic amino acid residues, while the other face is surrounded by a cluster of ordered water molecules (51, 52). The binding cavities of FABP3 and FABP4 are highly conserved showing a sequence identity of ca. 96% and only one mutated residue; namely Thr36 (FABP3) is mutated to Ala36 in FABP4. Meanwhile, the binding cavity of FABP5 shows a slightly higher divergence sharing ca. 73% and 77% sequence identity with FABP3 and FABP4, respectively. We docked 5- and 16-DSA into the binding cavity of FABP3 (PDB ID: 4WBK (48)) while keeping all the ordered water molecules inside the structure. Our docking results indicate that both derivatives can be accommodated into the binding cavity of FABP3 in a U-shaped conformation and show a good overlap with the observed binding modes of long-chain fatty acids. The carboxylate group shows salt-bridge interactions with the side chains of Arg106 and Arg126 in addition to H-bond interactions with Tyr128 and two ordered water molecules. In 5-DSA the DOXYL group is placed next to the ordered water cluster causing a slight shift of the position of the remaining fatty acid tail (Fig. 9). Meanwhile, in 16-DSA the DOXYL group is lined by the side chains of Thr36, Pro38, and Ser55 and the remaining fatty acid tail shows a high overlap with the co-crystallized stearic acid (Fig. S20).

#### Binding pocket polarity

The docking simulations showed the comparability of our model ligands used in the experiments with native stearic acid. This enables us to analyze the polarity of the binding pockets in more detail. The fluorescence intensity scans obtained by MST give first insights into the environment of ligands bound inside the β-barrel (see Fig. S2, *A*, *C*, and *E*). The intensity scans of the capillaries show a strong increase in fluorescence intensity for FABP5 with increasing protein concentration. This could be explained by a more hydrophobic environment of the bound fluorophore inside the binding pocket, leading to a higher quantum yield and higher fluorescence intensities. The same effect was already observed by Thumser *et al.* (53) for the fluorophore Bodipy Fl C16 in a mixture with FABP1 and FABP2. The relative polarity inside the binding pockets can very precisely be estimated by *a*_iso_ from the EPR simulations. The value decreases from free *via* intermediately bound to strongly bound components for FABP3 and FABP5. In the case of FABP4, the intermediately bound 5/16-DSA seems to be in an even more polar environment than free molecules. This seems to be unlikely, because polar and non-polar amino acid residues are clustered across the protein surface where the attached fatty acids are expected. Although the margin of error especially concerning the used *a*_iso_-values is rather high in FABP4 sample simulations, it should be noted that FABP4 exhibits a small cluster of polar amino acids opposite the portal region that could explain the polarity increase (see Fig. S21). The headgroups of attached 5/16-DSA molecules could interact, *e.g.*, with polar surface-exposed Lys and Arg residues near the portal region, or the alkyl chains could loosely interact with non-polar Phe residues. The large number of surface-exposed amino acid residues that are suitable for interactions precludes a clear statement about the nature of the attachment, but it may explain the high diversity of *a*_iso_ values for intermediately bound 5/16-DSA. Strongly bound 5/16-DSA is located in the more hydrophobic environment within the β-barrel, while free 5/16-DSA is fully exposed to the polar solvent. In the case of 5-DSA, the DOXYL group is located closer to the carboxylic acid headgroup and the polar amino acid residues, *for example*, Arg106, Tyr128 and Arg126 (Fig. 8*B*). Comparing the three FABPs, the biochemical nature of these three ‘anchoring’ residues within the binding pocket remains the same, only their sequence positions change. The closer contact with the hydrophilic amino acid residues and the simulated network of water molecules, salt bridges, and hydrogen bonds near the headgroup likely lead to a more polar environment at position C5 (54). This is indeed found in the larger value of *a*_iso_ for this spin probe, in contrast, 16-DSA reflects the environment near the ending of the alkyl chain with adjacent non-polar amino acid residues (see Figs. 8*D* and 9). When 5/16-DSA exhibit the simulated U-conformation, the DOXYL group could be oriented towards the alkyl chain or it could be exposed closer to the surrounding hydrophobic amino acid residues than 5-DSA, *e.g.*, to Ala75, Ser55, and Phe57. The latter is thought to bind the chain *via* hydrophobic interactions (55). This theory is supported by the slightly lower *a*_iso_ value of strongly bound 16-DSA in comparison to 5-DSA, indicating a less polar environment at position C16.

The simulations of the temperature series also indicate that the environment of the bound spin probe within the binding cavity becomes more hydrophobic with rising temperature, since the apparent *A*’_zz_ values of intermediately and strongly bound 5/16-DSA seem to decrease in some cases (see Fig. S19*B*). Theoretically, this observation could be explained by structural changes within the cavity, exchange of water, disruption of hydrogen bonds or closer contact with the Phe residues due to the higher dynamics of the ligand. However, a similar effect can be also approached by changes in the simulations of τ_c_ and *g*_iso_. Hence, the interpretation of this observation should be done with caution. The small decrease of *a*_iso_ for intermediately bound 16-DSA with higher concentrations of FABP3 (not shown) could arise from a higher protein density, generating a less polar environment for attached 16-DSA. Interestingly, the lowest *a*_iso_ values were found for 20/20 and 25/20. It should be noted that the changes discussed are small and that the simulations have some uncertainty. Including the results of our docking simulations, we cautiously interpret that van-der-Waals interactions of the alkyl chain with hydrophobic amino acid residues could play an important role in the orientation, the binding, and the conformation of the ligand within the β-barrel. In summary, we successfully utilized our radical bearing model ligands not only for spectroscopic binding studies but also as native-like polarity and geometry sensors, suitable for the amphiphilic binding pockets of proteins. Since we here combine EPR spectroscopy and molecular docking studies, we find the explicit effect of the DOXYL-group and the changes in the stearic acid backbone geometry expand the possibilities of binding studies beyond amphiphilicity, including conformational aspects of the ligand.

#### Protein hydropathy/polarity

In Figure 8*A* the protein surface is plotted and the clustering into alternating patches of hydrophilic and hydrophobic amino acids becomes visible. This structural property could be responsible for the solubility and could also be an affinity-determining factor for the binding of ligands or interactions with membranes. Hydropathy and polarity indices of the whole protein sequences were calculated and plotted in Fig. S21. As visualized, the assumed portal region combines polar and non-polar amino acid residues in a manner that facilitates the uptake of amphiphilic ligands. Total hydropathy indices of −33.7, −32.8, and −59.9 were calculated as the sum of the amino acid indices for FABP3, FABP4, and FABP5, respectively (see Fig. S21). Accordingly, FABP3 and FABP4 are more hydrophobic than FABP5, which could explain the lower solubility in the buffer and the stronger aggregation of the proteins at higher concentrations compared to FABP5. Despite the lower total polarity, FABP4 exhibits a polar surface cluster around amino acid position 112 opposing the portal region, as already mentioned before. However, EPR measurements do not show systematic polarity differences within the cavities of different isoforms. For example, 16-DSA bound by FABP5 shows lower *a*_iso_ values than FABP4, indicating that the overall hydrophobicity does not necessarily mirror the binding pocket hydrophobicity, but can also be related to the residues of the protein surface. This raises the question of the ‘evolutionary purpose’ of different surface polarities. An adaption to different tissue-specific milieus could be the reason for this observation. Adipose and skin tissues show water contents of 5 to 20% and 60 to 76%, respectively (56, 57). Therefore, a lower surface polarity would be thermodynamically more favorable in adipocytes. It has to be emphasized that FABPs are not limited to the single tissues they are named after and are often co-localized in different tissues. However, if the isoforms prefer different microenvironments inside the cells and operate synergistically with a higher adaptability to environmental changes, the different polarities of the FABPs could explain their co-localization in tissues. Further questions on the transferability of the previous results to real biological systems will be discussed in the following.

### Discussion of the results in the physiological context

How can the in-vitro, mainly spectroscopically achieved, results of this work be transferred to deliver insights into the biological functions of FABPs? Which conclusions—if any—can be drawn for the FABPs in native environments? For a comparison with the physiological conditions of the cytoplasm, a temperature of 37 °C and buffer/salt solutions with pH 7.5 were used, except for FABP4 which was handled in water. The pH value in the cytosol was found to be slightly lower with values of 7.0 to 7.2 (58). However, the value is also alternating in different areas of the cytoplasm, *e.g.*, at cell organelles (58), and is validated to be sufficiently exact. The highest FABP concentrations used in these studies were 200 μM. The physiological concentration within normal cytoplasm lies between 150 and 300 μM (12, 59, 60). Thus, the highest concentrated samples mirror physiological conditions most accurately. The physiological fatty acid concentration in cytoplasm is below 50 μM (59) which is also in accordance with the used 5/16-DSA concentrations of 20 μM. However, FABP and fatty acid distribution within cells can probably be seen as heterogeneous (61). The different FABP-to-5/16-DSA ratios cover a wide range of possible microenvironments, between tenfold protein excess (200/20), equal distribution (20/20), and fourfold fatty acid excess (5/20). Such microenvironments with locally higher or lower FABP or fatty acid concentrations could appear *e.g.*, at cell membranes, lipid droplets, vesicles, protein clusters, and cell organelles such as the nucleus and mitochondria. For the binding studies, model ligands were used which are chemically slightly different from native fatty acids. It is not fully clear, how the radical and fluorescent groups of the used fatty acids influence the binding affinity with the FABPs compared to non-labeled fatty acids. Influences of these groups on binding affinities compared to native fatty acids such as palmitic and stearic acid may be non-negligible. Even though the comparison of the binding behavior between labeled and non-labeled fatty acids under physiological conditions should be done with caution, the comparison between different FABPs is not affected by this circumstance, since the studies were performed consistently from the ligands’ viewpoints. Therefore, the trends and affinity rankings should be reliable and comparable to native systems. Furthermore, it can be stated with high certainty that the detected strongly bound molecules are located inside the binding pocket due to their slower dynamics and their less polar environment. These descriptions are based on and corroborated by the mainly spectroscopic data in this work. Additionally, it should be considered that the label groups of the fatty acids used to increase their solubility in water. This obviates the need for organic solvents such as ethanol or DMSO for pre-dissolving of the ligands and it should be noted that this is usually done for non-labeled long-chain fatty acids, too, and could also have a small impact on the results of previous binding studies (62, 63).

Different polarities and structural patches on the surface of FABPs could determine aggregation, fatty acid attachment, binding, and transport. Furthermore, the interaction with other proteins and membranes, the signaling processes and the possible directed diffusion could be controlled by these properties. At this point, it is questionable whether the observed protein aggregation is a physiological effect or only appears in the chosen model systems. Aggregation could disturb ligand transport, but could also facilitate it through ligand transfer based on protein collisions. The possible formation of dynamic clusters in highly concentrated solutions like the cytosol as an alternative diffusion mechanism has already been simulated and discussed in the literature (64). As discussed before, the FABPs show their highest activity at body temperature.

Easy binding, activation, and release require a low energy barrier and too strong binding would be inappropriate for the functionality of the FABPs. Therefore, the dominance of the intermediately bound fatty acids at 37 °C is not only consistent with the function of the FABPs within the cells but also sheds more light on the molecular origin as well as opens a door to thermodynamic analyses, which we can only touch here and which will be the topic in a subsequent project. The three FABPs show different binding affinities for saturated long-chain fatty acids and for themselves (self-aggregation). As suggested, a relationship between ligand binding and self-aggregation could exist in the cytoplasm or at the lipid membranes, either competitively as reported by Fournier *et al.* (35), or promotingly, as concluded from the results of this work. Aggregation could affect not only the binding affinity towards the fatty acids but also the activation and movement of the FABPs within the cytoplasm.

Finally, a closer look can be taken at the comparison between the three FABPs in the biological context. Similarities between FABP4 and FABP5 as opposed to FABP3 were found in different former studies with medical or biochemical focus. Both FABPs can be found in the same tissues and have similar effects on diseases and body functions, suggesting synergistic effects between them (11). Furthermore, the genes of both FABPs are co-localized on chromosome 8q21, whereas the FABP3 gene is located on chromosome 1p33-p31. The similar binding affinities and particle radius distribution patterns found for FABP4 and FABP5 in deviation to FABP3 could be loosely correlated with this biological information. However, the precipitation of FABP4 in the buffer could indicate a stronger aggregation depending on the medium used. In this context, FABP4 would act more similarly to FABP3, which could be related to the higher sequence similarity between the two FABPs. Although single amino acids were identified that could have an influence on the binding affinity, they could not yet be linked directly to the differences in the binding between the three FABPs. While the sequence identities are too low for specific amino acid analyses, at least larger structural elements, *e.g.*, patches with different polarities, may affect ligand binding and aggregation. Further studies and analyses need to be performed to better connect the found biophysical trends with biochemical and functional aspects within the human body.

### Conclusions and outlook

This study sheds new light on the mechanism, selectivity, and thermodynamic properties of fatty acid binding by FABPs. An in-depth view into these properties is required for a full understanding of their biological functions, cross-effects with other biomolecules, possible interactions with membranes, and their diverse roles in various diseases. It is likely that the FABP isoforms would not evolve without significant reasons. Even though the starting question about their evolutionary adaption remains partially unanswered, with our results obtained mainly from the perspective of the bound ligands, published information on FABPs can be expanded and re-evaluated and new insights into the function can be obtained. The main findings are summarized below.

(1) Firstly, the binding behaviors and affinities towards fatty acids are quite different for the three isoforms FABP3, FABP4, and FABP5. This phenomenon was partly known before and could now be confirmed by other methods. It supports the already existing hypothesis that the FABPs carry binding selectivity, genetically encoded within their sequences. FABP3 shows the highest affinity towards the fatty acids used here as confirmed by MST and EPR spectroscopy and simulations.
(2) Secondly, fatty acids may exhibit two different thermodynamic binding states when they are bound by FABPs. This was described for the first time here. The molecules can either be loosely attached to the protein, probably at its surface, or they can be bound strongly. The comparability of our utilized model ligands with native stearic acid could be verified *via* docking simulations. Hydrophobic interactions of the alkyl chain with amino acid residues within the binding pocket seem to play an important role for the strong binding of fatty acids. The intermediately bound state is interpreted as the thermodynamically most active state because it enables an easy uptake and release of the ligand. Therefore, it can be also seen as a transition or pre-binding state apparently dominant in physiological conditions. The theory of encoded binding selectivity may be expandable to the attachment of ligands as well.
(3) Furthermore, the FABPs show different temperature-dependent binding behavior. A temperature increase leads to the release of the ligand in the case of FABP3 and FABP4. In the case of FABP5 (and highly concentrated FABP3), the ligand is trapped within the binding pocket and at least the paramagnetic DOXYL group is possibly ‘inactivated’ during the denaturation of the protein. The binding of fatty acids appears to be thermodynamically optimized for physiological temperature.
(4) All FABPs aggregate at 37 °C within the buffer system used, but to varying degrees. FABP3 shows the highest and FABP5 has the lowest aggregation tendency. Self-aggregation is promoted by a temperature increase and in some cases by the attachment or binding of 5-DSA. However, for some FABP/5-DSA ratios, the monomeric or dimeric forms of the FABPs seem to be stabilized around the body temperature, and decreased aggregation after ligand addition was found, too.

The results should be further substantiated in the future by various studies. Our hypothesis of functionality adaption to the physiological temperature which we derived from the intermediate binding of ligands could be tested by studying FABPs from other species with different body temperatures, *for example*, *X. laevis* FABP1 or *C. elegans* LBP. Such a future project would possibly deliver important new knowledge about physical adaptions of proteins in general during the convergent evolution of species. Apart from the species dependence we are currently working on other human FABP isoforms, too, comparing disease-related point mutations of the same isoform to analyze if point mutations can also affect the binding and thermodynamics of the FABPs. More systematic analyses of how buffer/salt conditions and pH value influence the observed binding and aggregation behavior should be performed. The different binding states are currently further analyzed by pulsed EPR experiments with spin-probed or labeled FABPs in our lab. Finally, the research approach taken here can be also expanded to other FABPs and ligands. For example, binding analyses with (labeled) synthetic, pharmaceutically active, ligands could contribute a pharmaceutical standpoint. The presented results do not only expand the basic knowledge about FABP binding, but may also have corroborations as future biochemical and medical research method. In-depth knowledge about the thermodynamic and structural particularities of FABPs as amphiphilic transport proteins may be useful for the engineering of new artificial transport vehicles for directed drug transport in a biomimetic approach. Last but not least, our studies again confirm the power of simple spectroscopic experiments applied to biological model systems. The combined experimental approach used here will be further expanded in the future.

## Experimental procedures

### Materials

The human recombinant proteins FABP3, FABP4, and FABP5 were purchased from Sigma Aldrich (purity of ≥98% determined by SDS-PAGE by manufacturer). For additional studies on buffer-, pH- and aggregation dependence, native ligand displacement, and native gel analysis, new recombinant FABP4 (>95%) and FABP5 (>92%) were purchased afterward from antibodies-online.com. New FABP5 was desalted with the ÄKTA pure protein purification system (Cytiva) and lyophilized in water. Additional FABP3 with His-tag and 20% glycerol was purchased from Cayman Chemical (≥95%) for testing the comparability with the original pure and tag-free FABP3. The FABPs were reconstituted in ultrapure water supplied by a Milli-Q Advantage A10 (Millipore SAS, Merck), obtaining stock solutions of 210 μM. A buffer solution of 20 mM N-(2-Hydroxyethyl)piperazine-N’-(2-ethanesulfonic acid) (HEPES) and 150 mM sodium chloride (Sigma Aldrich, purity ≥99.5%), was prepared in ultra-pure water and set to a pH value of 7.5 with 0.1 M sodium hydroxide solution (Carl Roth GmbH & Co KG, purity ≥99%). FABP3 and FABP5 were diluted into HEPES/NaCl buffer solution with pH 7.5 to obtain sample concentrations suitable for EPR, MST, and DLS measurements (described below). In opposite to FABP3 and FABP5, FABP4 was diluted in ultrapure water, since salts and buffer were already contained from the manufacturing process. 5-DSA was purchased as an ammonium salt from Avanti Polar Lipids (Merck). It was dissolved in HEPES/NaCl buffer solution with pH 12, treated by ultrasonic for 10 min, and then diluted into HEPES/NaCl buffer with pH 7.5 to obtain a 0.5 mM stock solution. 16-DSA was obtained from Sigma Aldrich as an unmodified fatty acid. Unlike 5-DSA, it was dissolved in 0.1 M potassium hydroxide solution (Carl Roth GmbH & Co KG, purity ≥85%) at first and then treated similarly. The fluorophores rhodamine-PA and TopFluor-OA were purchased from Avanti Polar Lipids (Merck). Rhodamine-PA was dissolved in HPLC grade chloroform (Carl Roth GmbH & Co KG) to obtain a 616 μM stock solution, which was stored at −30 °C. From this solution, aliquots of 50 μl were taken with a Hamilton glass syringe and transferred into dark-toned glass vials. The chloroform was evaporated under a nitrogen stream and the residue was dissolved in 0.1 M KOH solution under 10 min of ultrasonication. It was then diluted in HEPES/NaCl buffer with pH 7.5 to obtain a 50 μM stock solution. TopFluor-OA was delivered as a 1 mg/ml chloroform solution, from which aliquots of 20 μl were transferred into glass vials. After evaporating the chloroform, the residue was dissolved in HPLC-grade ethanol (Carl Roth GmbH & Co KG) to obtain a 100 μM solution. An aliquot of the ethanol solution was transferred into HEPES/NaCl buffer solution, yielding a 1 μM aqueous stock solution with a final ethanol concentration of less than 0.1%. Tris(hydroxymethyl)aminomethane hydrochloride (TRIS-HCl/NaCl) solution (Carl Roth GmbH & Co KG, purity ≥99%) was prepared similarly to HEPES/NaCl and set to pH 7.5. Basic and acidic HEPES/NaCl solutions were prepared by adjusting the pH value with sodium hydroxide and hydrochloric acid (Carl Roth GmbH & Co KG). Cis-4,7,10,13,16,19-Docosahexaenoic acid (DHA) (Sigma Aldrich, ≥98%) was dissolved in ethanol and diluted into HEPES/NaCl for a final ethanol concentration of less than 0.1%. All substances were used without further purification.

### CW EPR spectroscopy

CW EPR spectroscopic measurements were performed at X-band frequencies (∼9.4 GHz) with a benchtop spectrometer Magnettech MS-5000 (Freiberg Instruments GmbH, now Bruker Biospin). The samples were prepared in low-binding PCR tubes (250 μl). Protein and 5/16-DSA solutions were mixed with HEPES/NaCl buffer at pH 7.5, to obtain samples with a constant 5/16-DSA concentration of 20 μM and varying FABP concentrations of 200, 100, 50, 35, 25, 20, 15, 10 and 5 μM. After an incubation time of 20 min, the samples were transferred into glass capillaries, which were sealed with ‘Critoseal’. At first, solutions with 20 μM of 5/16-DSA were measured as references at 37 °C. The concentration series of FABP with 20 μM 5/16-DSA were measured at 37 °C, directly after preparation. Afterward, the sample capillaries were stored at 4 °C, and subsequent temperature-dependent measurements were performed. They were carried out as temperature series from −30 °C to 90 °C with stepwise measurements every 10 or 5 °C and a final measurement again at 37 °C. All measurements consisted of 10 scans, each scan with 60 s of measuring time. The field sweep was set from 332.5 to 342.5 mT with a modulation amplitude of 0.1 mT and a modulation frequency of 100 kHz. A microwave power of 4.79 mW was used. For additional buffer- and aggregation-dependent measurements and the DHA displacement study the same parameters were used, only the concentrations were adjusted as described before.

### Analysis of EPR spectroscopic data and spectroscopic simulations

For plotting the measured spectra and the EPR spectral simulations, MATLAB (version 9.11.0.1809720, R2021b) and the Easyspin package (version easyspin-6.0.0-dev.43) (65) were used. The Easyspin function ‘Chili’ for CW EPR spectra in the slow-motional regime as developed by Schneider and Freed was applied (65, 66) and three spectral components 1, 2 and 3 were simulated. For all systems, an electron spin of S = 1/2 was set. By calculating the double integrals of the components, their proportions were simulated. The error bars for simulated component proportions (Φ) in binding curves were estimated by using the root-mean-square deviations (RMSD) of the simulations as absolute errors (Φ ± RMSD%). It should be noted that this strategy is an estimation, since the contributing partial errors of simulated parameters *a*_iso_ and *t*_c, iso_ are neglected for simplification. Errors of *K*_D_ and Δ*G* values derived from the simulations were estimated (in cases where *K*_D_ was read out from the binding curves) or calculated *via* error propagation. The simulated values of the Landé factors *g* and the rotational correlation times τ_c_ are given as mean, isotropic values in Table S1, since no significant changes were made for the tensor values. The apparent hyperfine coupling constant *A*′ is given with all tensor values *A*’_xx_, *A*’_yy_, *A*’_zz_, although most often only *A*’_zz_ was changed. In the main part of this work, only changes of the isotropic values τ_c, iso_ and *a*_iso_, as well as of *A*’_zz_ will be discussed. The relative errors of τ_c_ were estimated as ± 10% for (1) and ± 5% for (2) *via* trial-and-error, while the errors of *A*’_zz_ and *a*_iso_ were similarly estimated as ± 1%. The parameter values were adjusted for different isoforms, FABP concentrations, spin probes, and temperatures. Small adjustments of *g*_xx_, *g*_yy_, *g*_zz_, *A*’_xx_, *A*’_yy_ were only done if necessary and will not be discussed here (see also Table S1). Additional Gaussian linewidth of 0.13 to 0.14 was simulated for component (1) and Lorentzian linewidth of 0.1 for component (3). The Euler angles α, β and γ were simulated to be 0°, 50° and 0°. Finally, for component (2), Heisenberg spin exchange broadening of 2.7 MHz was simulated, while it was held at 0 MHz for component (1) and (3).

### Microscale thermophoresis

Microscale thermophoresis (MST) experiments were carried out on a Monolith NT.115 MST device (NanoTemper Technologies GmbH). For measurements with rhodamine-PA a green excitation filter (510–550 nm) and for TopFluor-OA a blue filter (465–490 nm) was used. All measurements were done at 25 °C. In order to minimize the effect of protein aggregation, 0.05 Vol-% of *Tween 20* (NanoTemper Technologies GmbH) were added to the HEPES/NaCl buffer solution. Samples were prepared in low-binding Eppendorf tubes. The influence of photobleaching on the quantum yield was reduced by using freshly diluted fluorophore solutions and storing the samples under the exclusion of light during the incubation time of 10 min. The samples were loaded into premium capillaries (NanoTemper Technologies GmbH). The coated capillary surface is designed to avoid the adhesion of molecules. Initially, repeated pre-tests without protein but with different fluorophore concentrations were carried out. This was done to find the optimum concentration and excitation power and to check the fluorescence activity of the fluorophores. The fluorescence intensity had to be in the range of 300 to 600 counts, with preferably smooth MST traces. For binding affinity measurements, the concentration of the fluorescent fatty acid was held constant at 80 nM, while the non-labeled FABPs were used at variable concentrations in a dilution series. The power of the IR laser was held constant at a medium set for all measurements, generating a constant temperature gradient. The measurements were performed as duplicates, using the software MO. Control (version 1.6) and afterward analyzed with the software MO. Affinity Analysis (version 2.3) (NanoTemper Technologies GmbH). The mean fluorescence intensities at the start time were divided by the values at the end time of the MST traces and normalized, obtaining the MST signal *F*_norm_. For all analyses, the end time was manually set to 1.5 s after the start of the heating process. *F*_norm_ was normalized for the amplitude and baseline corrected to obtain the bound fraction. This was plotted against the ligand concentration and error bars were calculated by standard deviation. The data points were fitted by using a K_D_ model for 1:1 interaction, the *K*_D_ value was extracted and a confidence range (in %) for the value was calculated, assuming 68% of confidence.

### Dynamic light scattering

Dynamic light scattering was carried out with a Lightsizer 500 device from Anton Paar (Graz, Austria). A wavelength of 658 nm and the setting ‘side scatter’ were used. The latter detects the light at an angle of 90° relative to the incident beam direction. Measurements were performed as temperature series, starting at 37 °C, cooling down to 0 °C and gradually heating up to a temperature of 90 °C, with a final measurement again at 37 °C. The measurements were done automatically every 5 °C or 10 °C. An equilibration time of 2 min and a measurement time of 30 s was set, measuring 6 runs. A quartz low-volume cuvette was filled with 80 μl of the sample solution and closed with a lid to avoid the evaporation of water at high temperatures. The cuvette was cleaned with ultrapure (resistance larger 18.6 MOhms) water, ethanol, and acetone (Carl Roth GmbH & Co KG) after every measurement and dried within a jet of clean nitrogen. The autocorrelation curves obtained from the DLS measurements were baseline corrected and manually fitted using the ALV-Correlator software (version 3.0) from ALV-GmbH. An exponential fit model was used. The lower distribution radius was set to 0.5 nm and the upper radius was set to 2500 nm. The number of grids was set to the maximum value of 250.

### Native and sodium dodecyl sulfate (SDS)–polyacrylamide gel electrophoresis (PAGE)

The blue native gel preparation (see Supporting information), loading, and running were performed at 4 °C. The samples containing 20/0, 20/10, 20/20, 20/50, 20/100, 50/0, 50/20 and 50/50 FABP4/16-DSA were diluted into the loading buffer and 10 μl were loaded into the gel pockets without prior heating. A molecular size marker (3 μl) was loaded two times into the outer gel pockets. The gel was running at 90 V for 4.5 h. Afterward, the gel was decolorized overnight under shaking and scanned for analysis. For the SDS-PAGE the same samples were diluted into a loading buffer containing SDS and β-mercaptoethanol. Additionally, the samples were heated to 95 °C for 5 min. It was used a 10% SDS PAGE gel with 0.1% SDS in the running buffer. The gel was running for 55 min at 240 V. The same molecular marker as for the native gel was used.

### Structural analyses

The amino acid sequences and the tertiary structures of FABP3, FABP4, and FABP5 in their apo-forms as well as complexed with stearic acid, were obtained from the *Research Collaboratory for Structural Bioinformatics* (RCSB) protein databank (PDB) (13, 48, 50). The structures were plotted using the software PyMol (version 2.5.2) by Schrödinger, LLC and colored according to the secondary structural elements and the amino acid hydrophobicity, using the scale of Eisenberg (67). Sequence alignments were achieved with the Expasy SIM Alignment Tool (68). Hydropathy indices were calculated and plotted with ProtScale (69), applying the scale developed by Kyte and Doolittle (49). The total hydropathy indices of the FABPs were calculated as the sum of all amino acid indices within the sequence. For polarity plots, the scale of Zimmermann (70) was used.

### Docking studies

The structures of 5-DSA and 16-DSA were generated in MOE [Molecular Operating Environment (MOE 2019.01; Chemical Computing Group Inc, 1010 Sherbooke St West, Suite #910, Montreal, QC, Canada, H3 A 2R7, 2019) in the N-oxide and carboxylate form. The ligands were subsequently prepared for docking using the LigPrep tool as implemented in the Schrödinger software (version 2021.3) without allowing the alteration of the ionization state. Energy minimization was performed using the OPLS4 force field. 25 conformers of the ligands were subsequently generated using ConfGen.

The X-ray structure of FABP3 in complex with stearate (PDB ID 4WBK) was fetched into Maestro (Schrödinger version 2021.3) from the Protein Data Bank (PDB; www.rcsb.org). Protein preparation was performed using the protein preparation wizard implemented in Schrödinger version 2021.3 by adding hydrogen atoms, assigning the protonation states, and minimizing the protein using the OPLS4 force field. All ordered water molecules were kept for the subsequent docking studies.

Docking of the generated conformers was performed using Glide (Schrödinger version 2021.3) in the Standard Precision (SP) mode while using enhanced sampling for the ligand conformation. The conformational sampling was enhanced by 4 times.

The docking setup was validated by redocking stearate into the binding cavity of FABP3. To avoid bias, the structure of stearate was regenerated from the respective SMILES, and the ligand was prepared as previously described for 5- and 16-DSA. The top-scored docking pose showed an RMSD of 1.1 Å with respect to the co-crystallized stearate.

## Data availability

Raw data can be provided by the authors on request.

## Supporting information

This article contains supporting information.

## Conflict of interest

The authors declare that they have no conflicts of interest with the contents of this article.
