# Supplementary material for: Fatty acid binding to the human transport proteins FABP3, FABP4, and FABP5 from a Ligand’s perspective
Source: J Biol Chem. 2024 May 20;300(6):107396. doi: 10.1016/j.jbc.2024.107396 (PMC11231610; doi:10.1016/j.jbc.2024.107396)
Supplement: Supporting information [file mmc1.pdf]

# Supporting Information

## Fatty Acid Binding to the Human Transport Proteins FABP3, FABP4, and FABP5 from a Ligand's Perspective

Sebastian Michler[1], Florian Arndt Schöffmann[1], Dina Robaa[2], Jonas Volmer[1] and Dariush Hinderberger[1,*]

[1]*Martin Luther University Halle-Wittenberg, Institute of Chemistry, Physical Chemistry – Complex Self-Organizing Systems, Von-Danckelmann-Platz 4, 06120, Halle (Saale), Germany*

[2]*Martin Luther University Halle-Wittenberg, Institute of Pharmacy, Department of Medicinal Chemistry, Kurt-Mothes-Straße 3, 06120 Halle (Saale), Germany*

**to whom correspondence should be addressed: dariush.hinderberger@chemie.uni-halle.de*

*Results of the MST measurements*

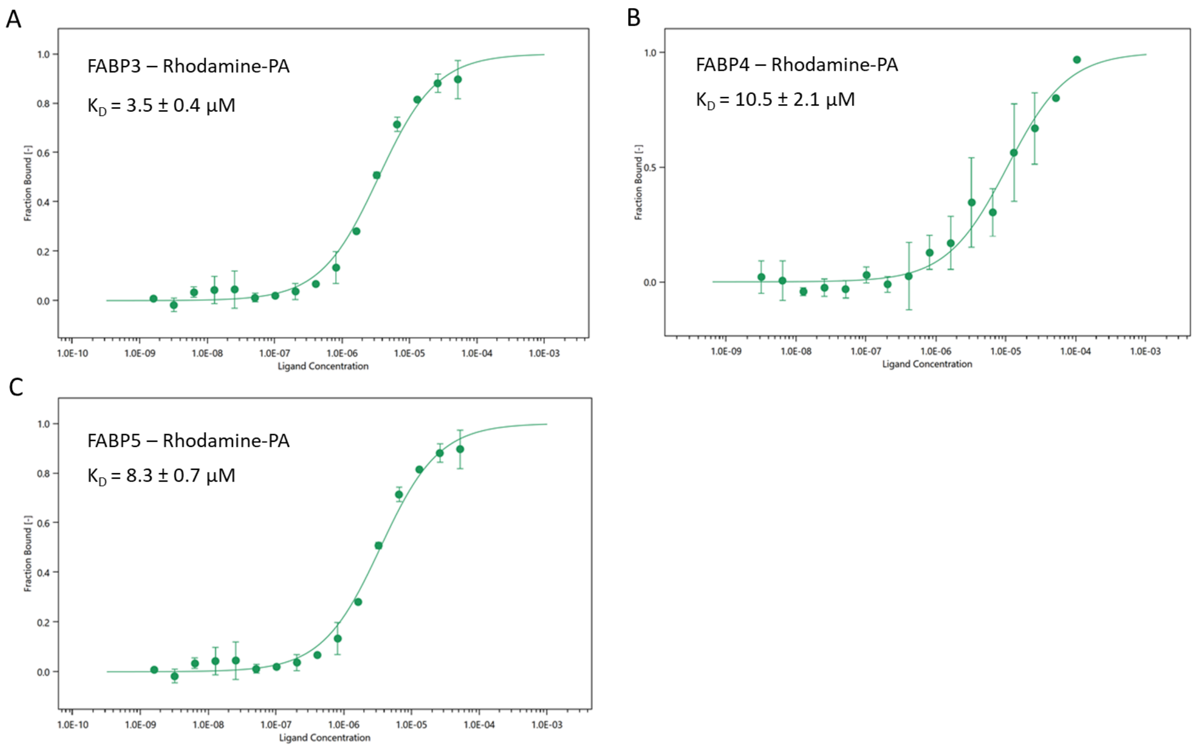


**Figure S1**: Binding curves for rhodamine-PA with FABP3 (A), FABP4 (B) and FABP5 (C) calculated from two merged MST affinity measurements for each FABP. The FABP (here: the ligand with the concentration in M) was titrated against 80 nM of rhodamine-PA. A starting protein concentration of 52.5 µM with an excitation power of 20 %, 105 µM with 100 % and 52.5 µM with 40 % were used for FABP3, FABP4 and FABP5, respectively.

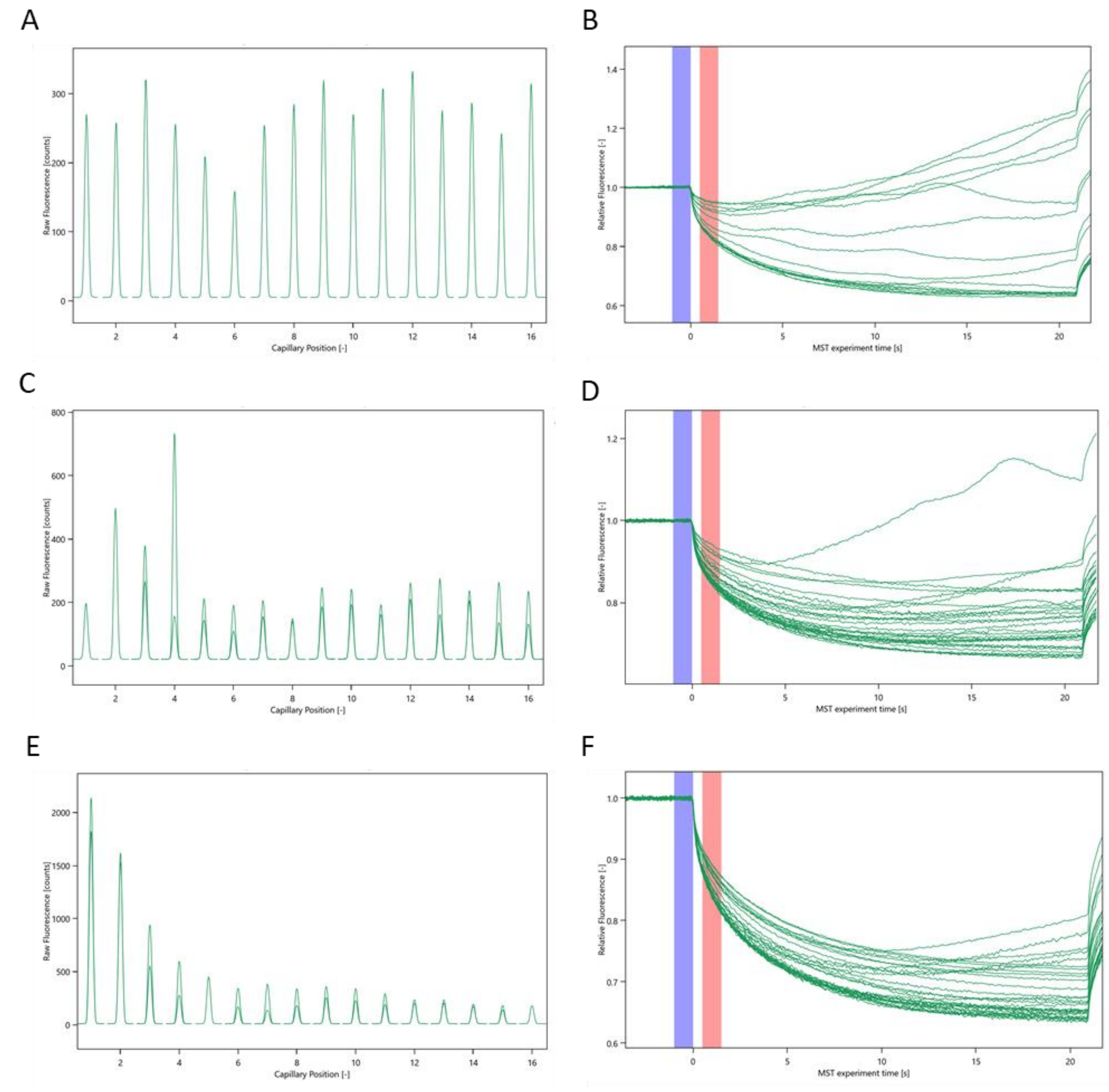


**Figure S2**: MST capillary intensity scans (left) and MST traces (right) of rhodamine-PA with FABP3 (A-B), FABP4 (C-D) and FABP5 (E-F).

*Concentration-dependent CW EPR spectra*

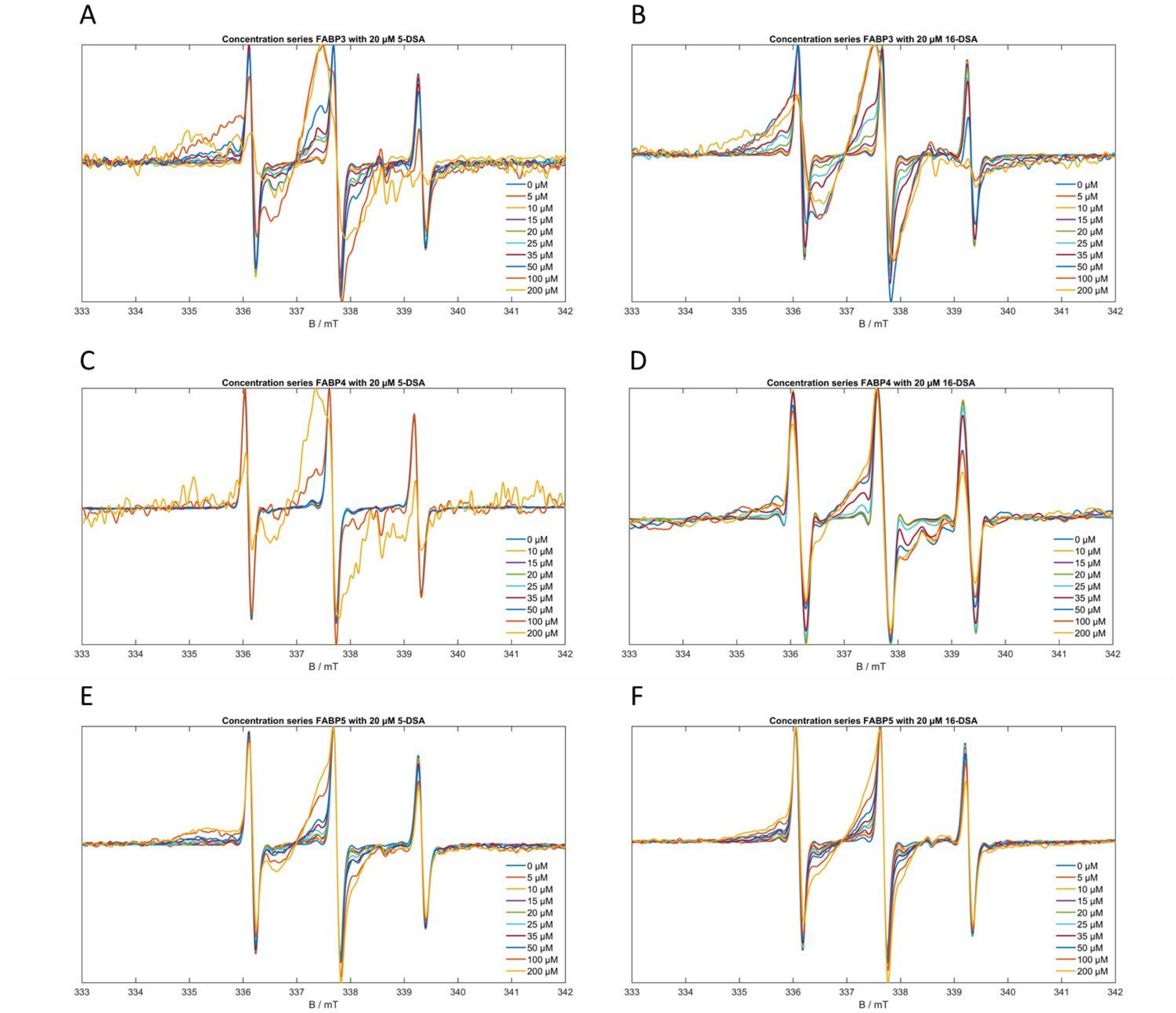


**Figure S3**: Intensity-normalized CW EPR spectra of 20 µM 5- and 16-DSA with concentration series of FABP3 (A-B), FABP4 (C-D) and FABP5 (E-F). The linewidth of the spectra was increased artificially.

*EPR simulations of the concentration series*

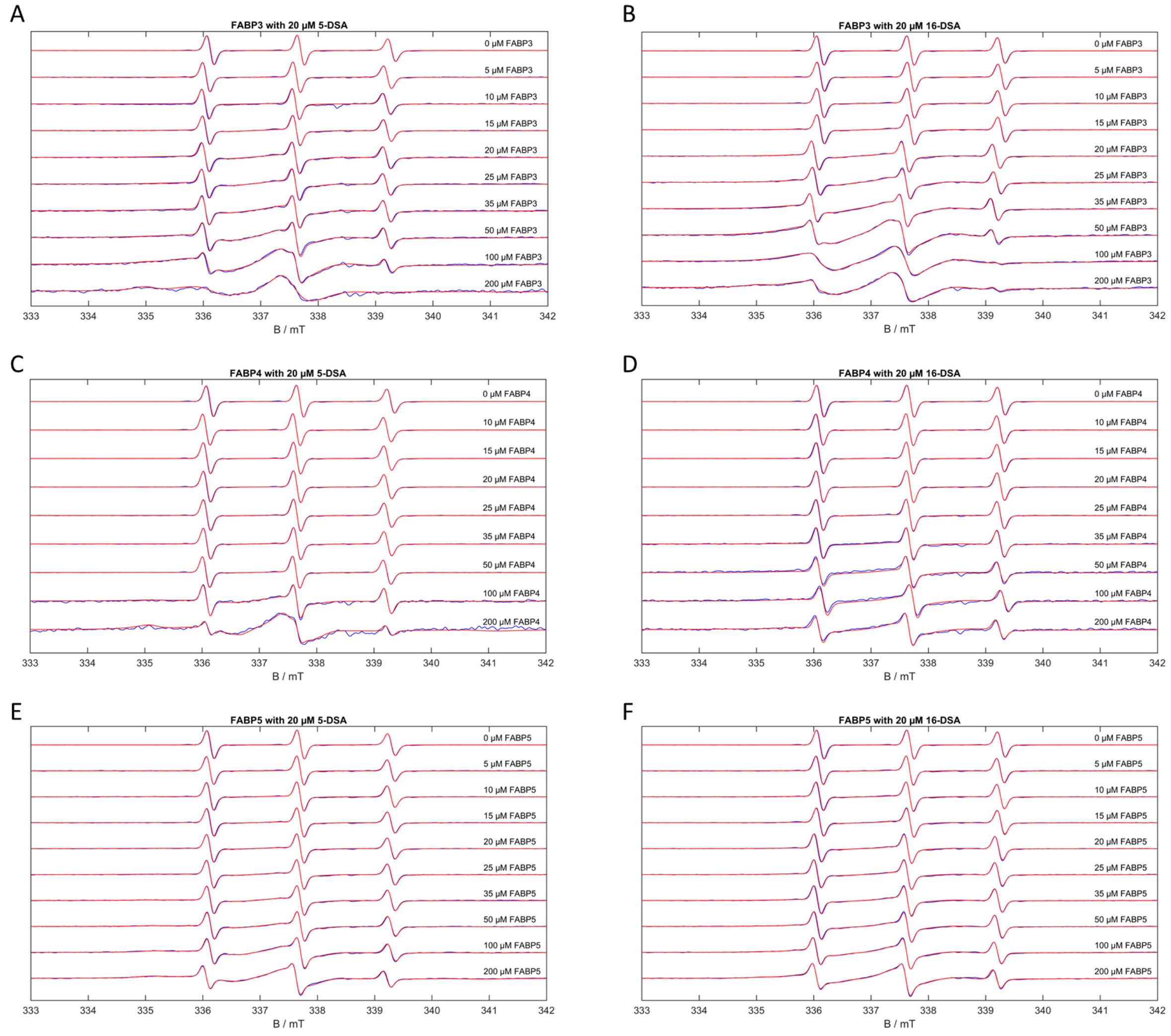


**Figure S4**: CW EPR spectra (blue) and simulations (red) of 5/16-DSA with concentration series of FABP3 (A-B), FABP4 (C-D) and FABP5 (E-F). The linewidth of the spectra and simulations were increased artificially.

*Parameters of the EPR simulations*

**Table S1:** Simulated EPR parameters of components (1), (2) and (3) for 5/16-DSA with FABP3.

| 5/16-DSA | | $g_{iso}$ | $a_{iso}$ [MHz] | $\tau_{c,iso}$ [ns] | $A'_{xx}$ [MHz] | $A'_{yy}$ [MHz] | $A'_{zz}$ [MHz] |
|---|---|---|---|---|---|---|---|
| 5-DSA | (1) | 2.0053 | 44.1 | 0.1 | 13.2 | 12.9 | 104.2 |
| | (2) | 2.0055 | 42.9 | 4.8-4.9 | 13.7 | 13.9 | 100.2 |
| | (3) | 2.0055 | 41.3 | 17.9-19 | 13.0 | 13.0 | 98.0 |
| 16-DSA | (1) | 2.0052 | 44.1 | 0.04-0.1 | 13.2 | 12.9 | 104.2 |
| | (2) | 2.0053 | 42.2 | 8.4-5.2 | 11.7 | 16.9 | 98.2 |
| | (3) | 2.0054 | 38.7 | 22.2-19.1 | 10 | 10 | 96.0 |

**Table S2:** Simulated EPR parameters of components (1), (2) and (3) for 5/16-DSA with FABP4.

| 5/16-DSA | | $g_{iso}$ | $a_{iso}$ [MHz] | $\tau_{c,iso}$ [ns] | $A'_{xx}$ [MHz] | $A'_{yy}$ [MHz] | $A'_{zz}$ [MHz] |
|---|---|---|---|---|---|---|---|
| 5-DSA | (1) | 2.0051 | 44.1 | 0.08-0.06 | 15.2 | 12.9 | 104.2 |
| | (2) | 2.0051 | 46.9 | 4.8 | 13.7 | 14.9 | 112.2 |
| | (3) | 2.0053 | 43 | 17.9 | 18.0 | 18.0 | 93.0 |
| 16-DSA | (1) | 2.0049 | 44.7-48 | 0.05-0.13 | 13.2 | 12.9 | 104.2 |
| | (2) | 2.0053 | 42.2 | 5.8-4.0 | 17.0 | 17.0 | 100.2 |
| | (3) | 2.0047 | 36.7-45.3 | 22.2 | 10 | 10 | 90.0 |

**Table S3:** Simulated EPR parameters of components (1), (2) and (3) for 5/16-DSA with FABP5.

| 5/16-DSA | | $g_{iso}$ | $a_{iso}$ [MHz] | $\tau_{c,iso}$ [ns] | $A'_{xx}$ [MHz] | $A'_{yy}$ [MHz] | $A'_{zz}$ [MHz] |
|---|---|---|---|---|---|---|---|
| 5-DSA | (1) | 2.0049 | 44.1 | 0.09-0.1 | 15.2 | 12.9 | 104.2 |
| | (2) | 2.0052 | 46.9-44.9 | 4.8 | 13.7 | 14.9 | 112.2 |
| | (3) | 2.0054 | 43.7-42.3 | 17.9 | 17 | 17 | 96 |
| 16-DSA | (1) | 2.0051 | 44.1 | 0.05-0.07 | 15.2 | 12.9 | 104.2 |
| | (2) | 2.0054 | 42.3-41.6 | 5.7 | 15.9 | 16.9 | 94.2 |
| | (3) | 2.0054 | 37 | 22.2 | 10 | 10 | 93 |

## *Concentration-dependent binding curves*

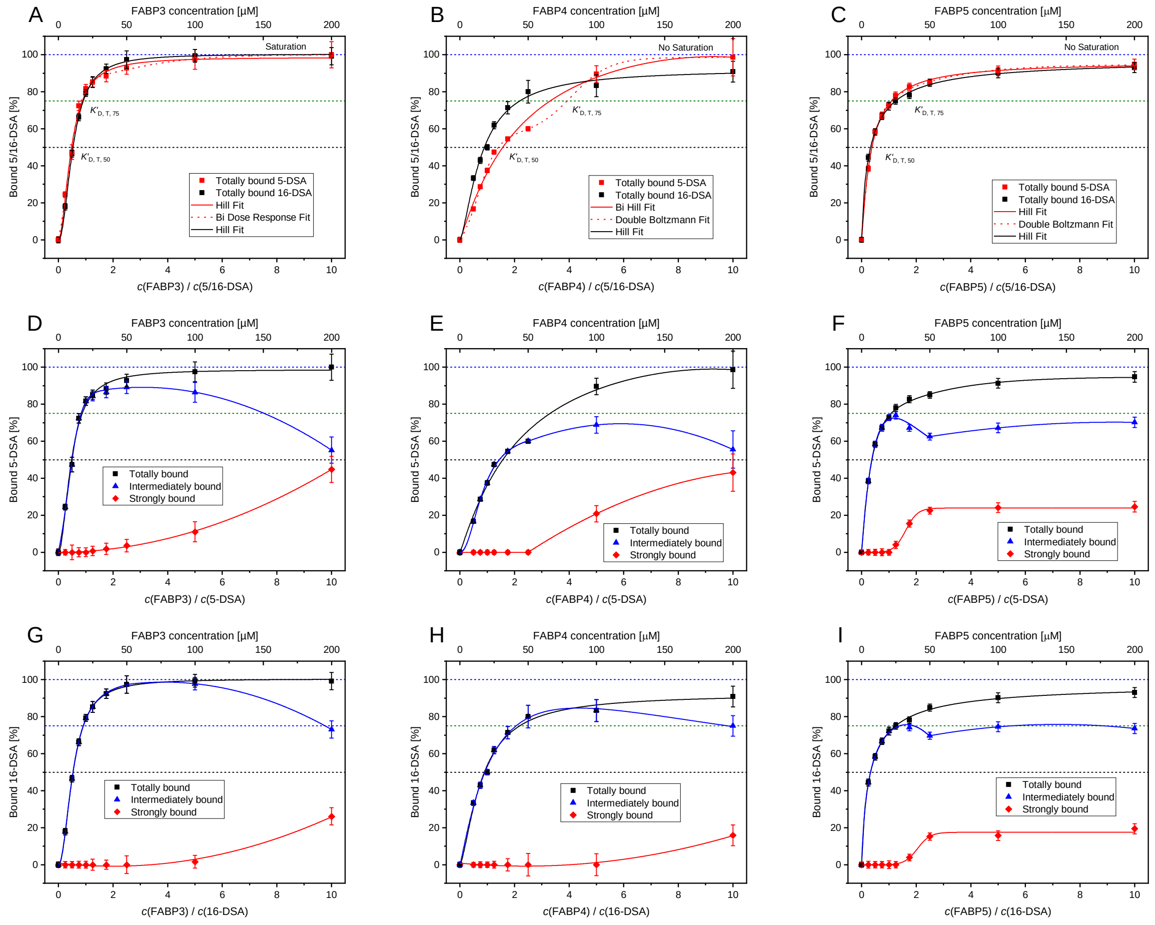


**Figure S5**: Binding curves derived from the double integrals of simulated spectral EPR components. A-C: Curves of totally bound 5/16-DSA with FABP3/4/5, D-F: Separated curves of 5-DSA with FABP3/4/5, G-I: Separated curves of 16-DSA with FABP3/4/5.

*Buffer and pH dependence*

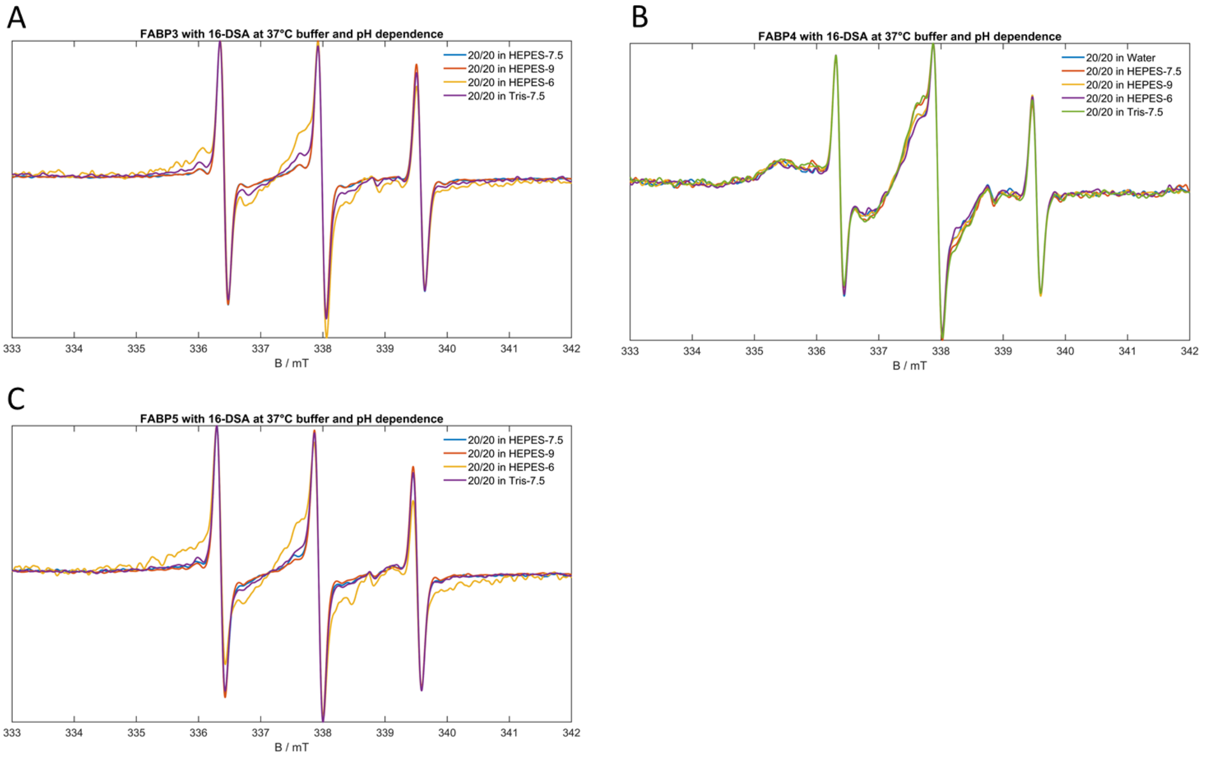


**Figure S6**: Intensity-normalized CW EPR spectra of 20 µM 16-DSA with 20 µM FABP3 (A), FABP4 (B) and FABP5 (C) in HEPES at pH 7.5, 6 and 9, in TRIS at pH 7.5 and in water (only FABP4). The linewidth of the spectra was increased artificially.

*DHA displacement*

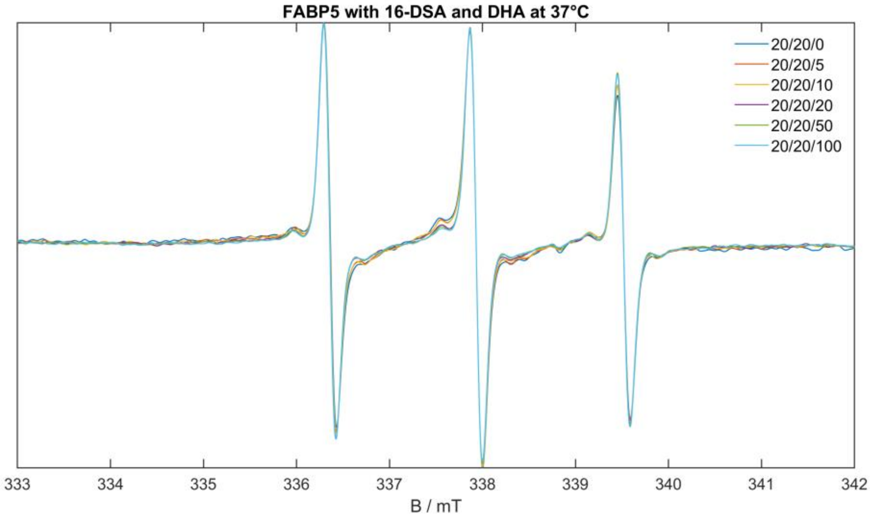


**Figure S7**: Intensity-normalized CW EPR spectra of 20 µM 16-DSA with 20 µM FABP5 and 0, 5, 10, 20, 50 and 100 µM DHA in HEPES/NaCl at pH 7.5.

*Temperature-dependent CW EPR spectra and simulations*

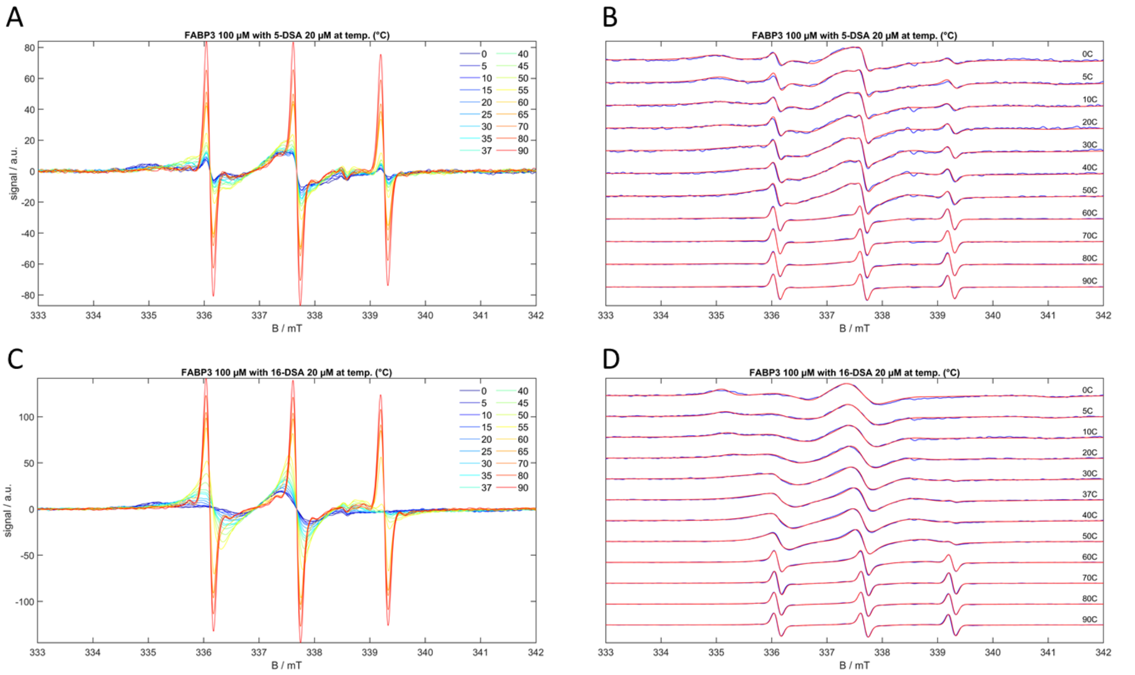


**Figure S8**: Temperature-dependent CW EPR spectra series (left) and stacked spectra (right, blue) with simulations (right, red) of 100 µM FABP3 with 5-DSA (A-B) and 16-DSA (C-D).

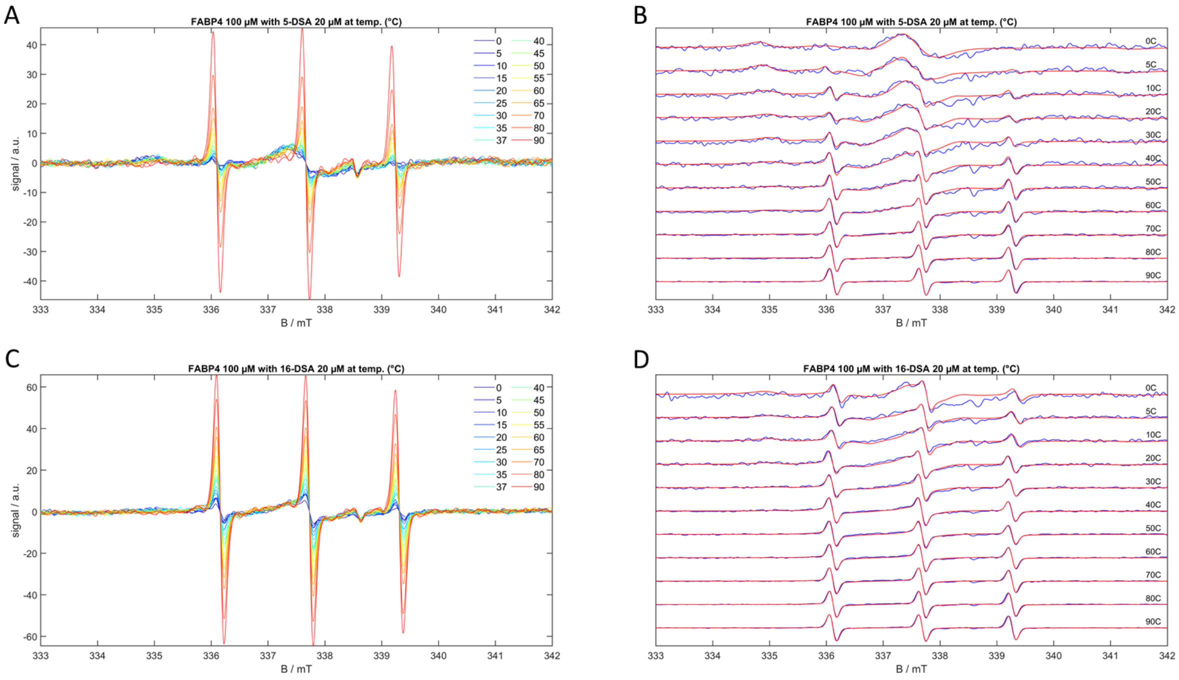


**Figure S9**: Temperature-dependent CW EPR spectra series (left) and stacked spectra (right, blue) with simulations (right, red) of 100 µM FABP4 with 5-DSA (A-B) and 16-DSA (C-D). The simulations are only approximated for low temperatures and not fully accurate due to low signal-to-noise ratios of the measured spectra.

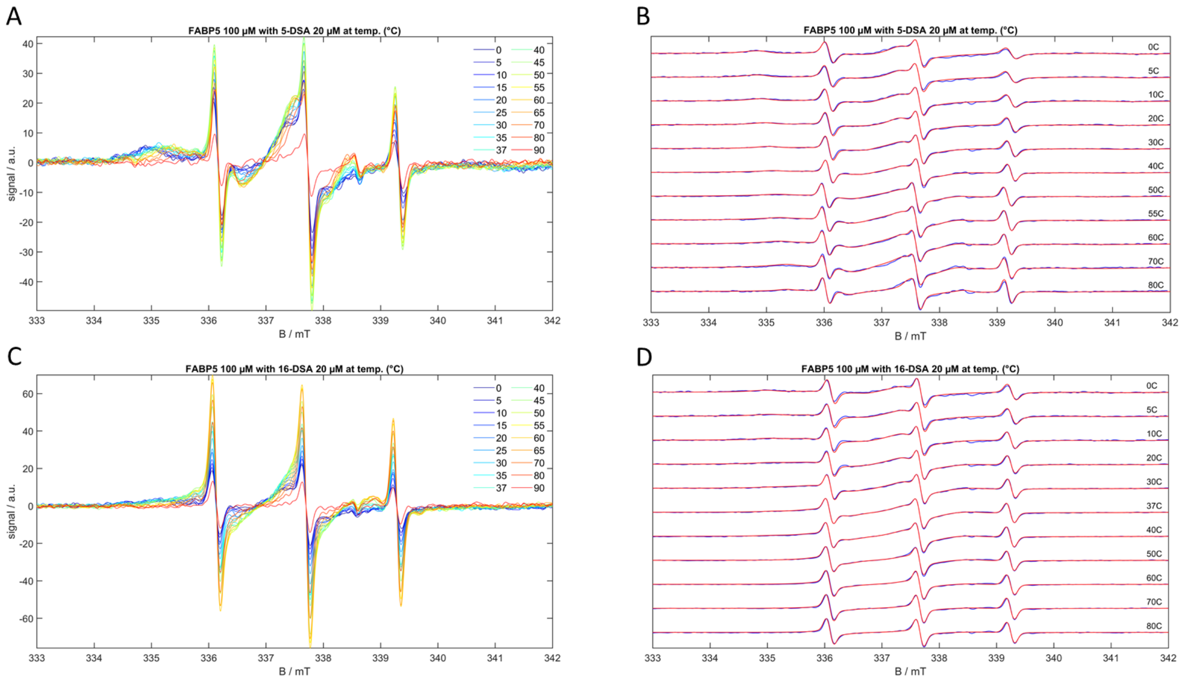


**Figure S10**: Temperature-dependent CW EPR spectra series (left) and stacked spectra (right, blue) with simulations (right, red) of 100 µM FABP5 with 5-DSA (A-B) and 16-DSA (C-D).

*Supporting CW EPR spectra series without simulations*

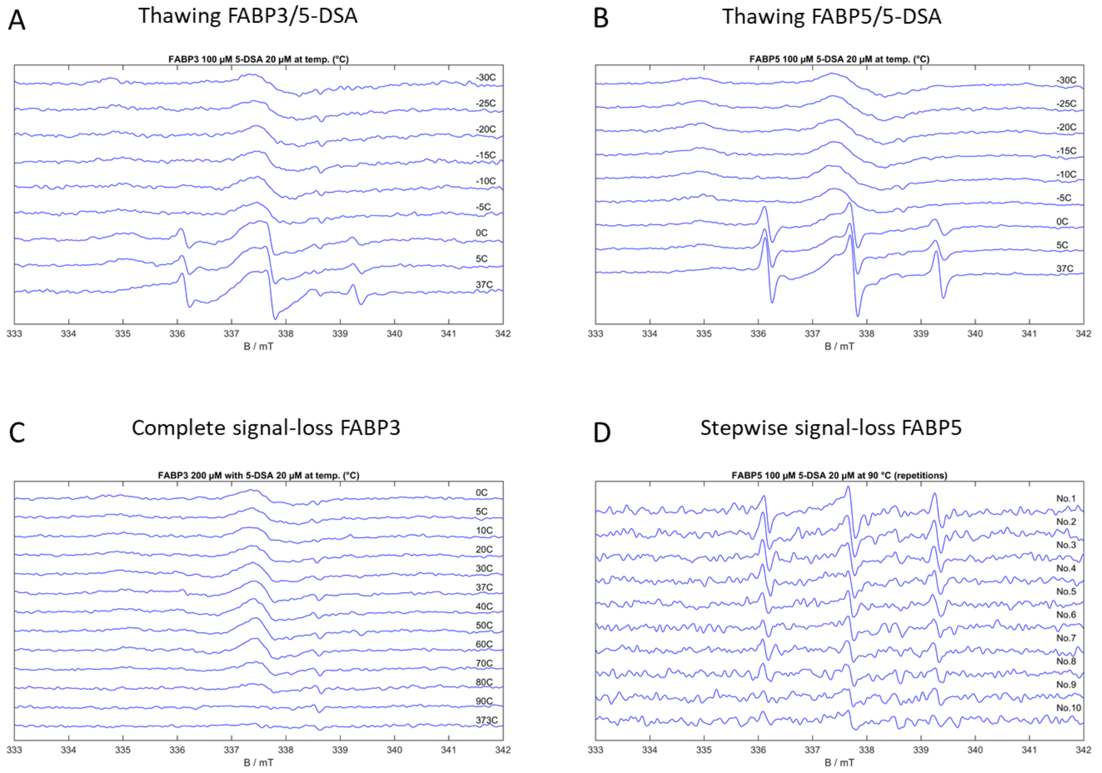


**Figure S11**: Additional temperature-dependent spectra series. A: Thawing of a FABP3/5-DSA mixture. B: Thawing of a FABP5/5-DSA mixture. C: Complete signal-loss of FABP3/5-DSA 200/20 at high temperature. D: Repetitive scans of FABP5/5-DSA 100/20 at 90 °C with stepwise signal loss.

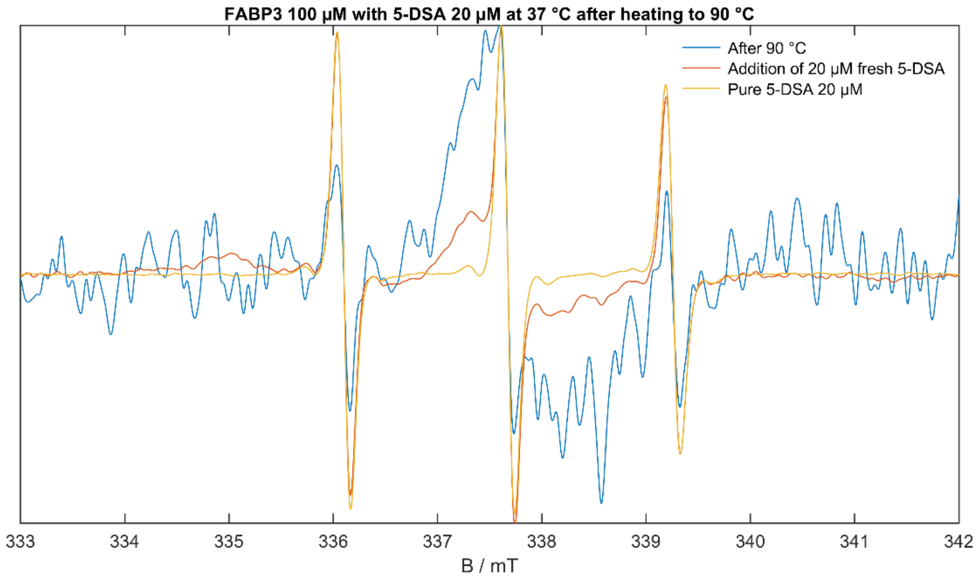


**Figure S12**: Binding behavior of FABP3 at 37 °C after heating up to 90 °C. CW EPR spectra of FABP3/5-DSA 100/20 without additional 5-DSA (blue), with additional, fresh 20 µM 5-DSA (red) and reference spectrum of pure 20 µM 5-DSA.

## *Supporting temperature-dependent binding curves*

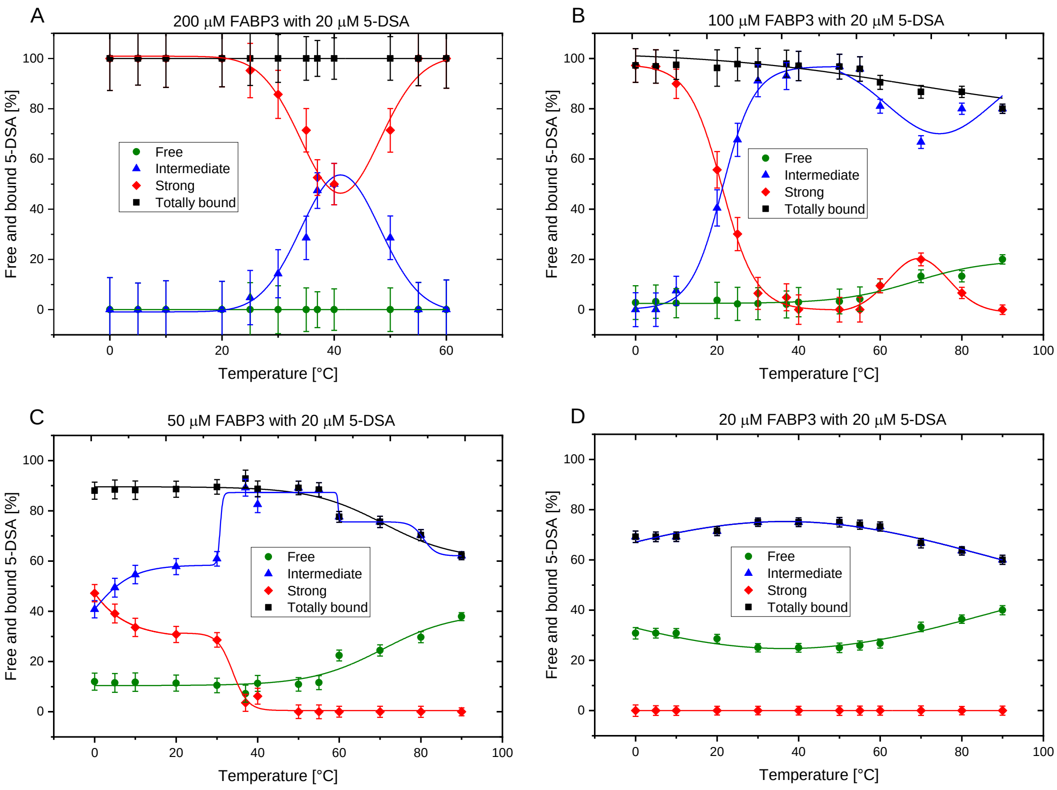


**Figure S13**: Temperature-dependent binding curves of 20 µM 5-DSA with 200 (A), 100 (B), 50 (C) and 20 µM (D) FABP3.

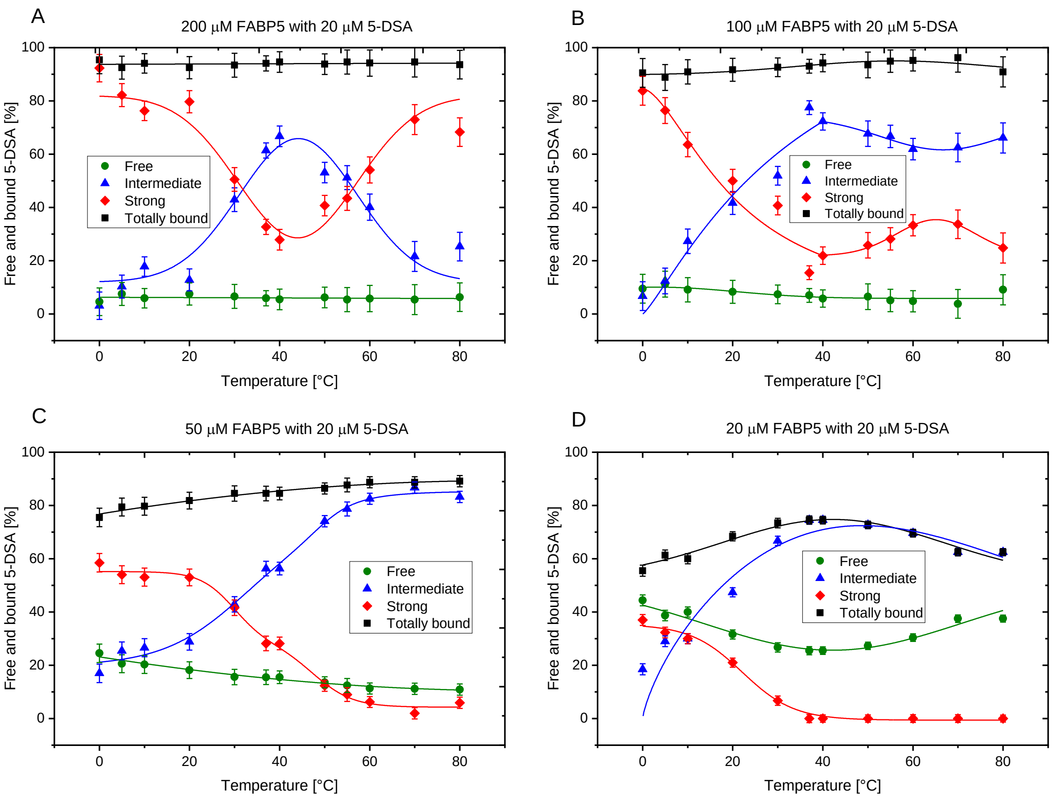


**Figure S14**: Temperature-dependent binding curves of 20 µM 5-DSA with 200 (A), 100 (B), 50 (C) and 20 µM (D) FABP5.

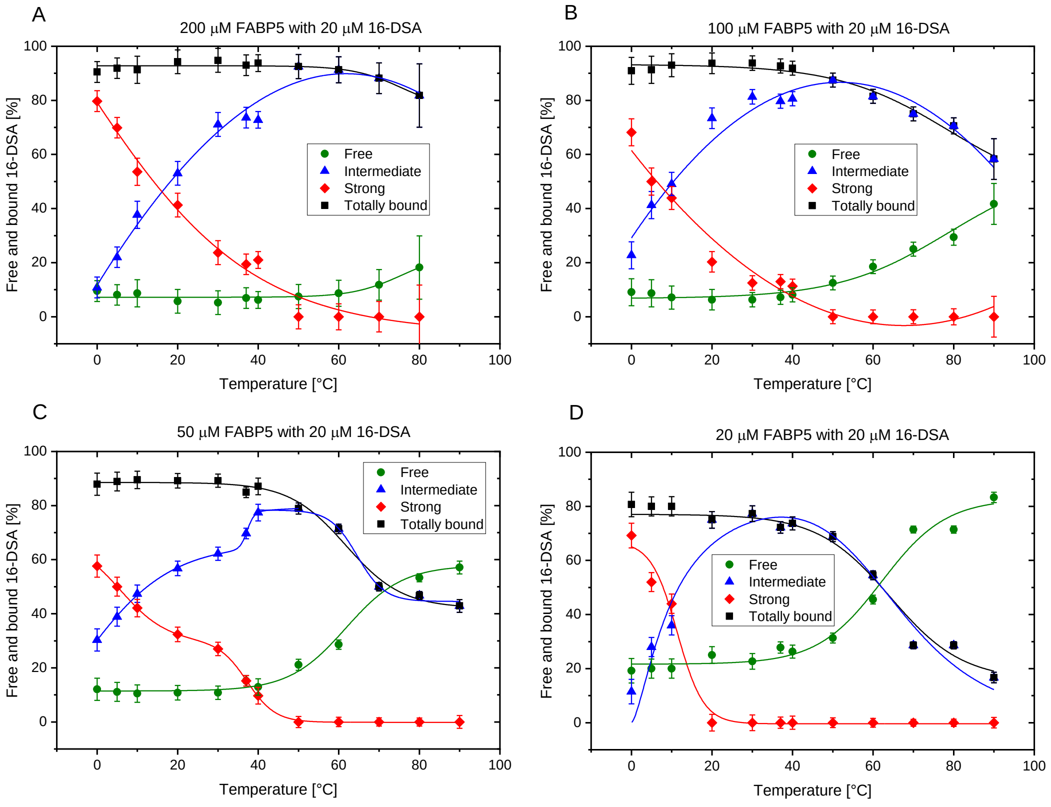


**Figure S15**: Temperature-dependent binding curves of 20 µM 16-DSA with 200 (A), 100 (B), 50 (C) and 20 µM (D) FABP5.

**Comment:** The solid lines within the temperature-dependent binding curves were produced by several different fit functions, but serve only as guide-to-the-eye and contain no physical information here. Hence, the fit equations are not depicted in this work, but can be provided on request.

*Aggregation/binding relationship in CW EPR spectra*

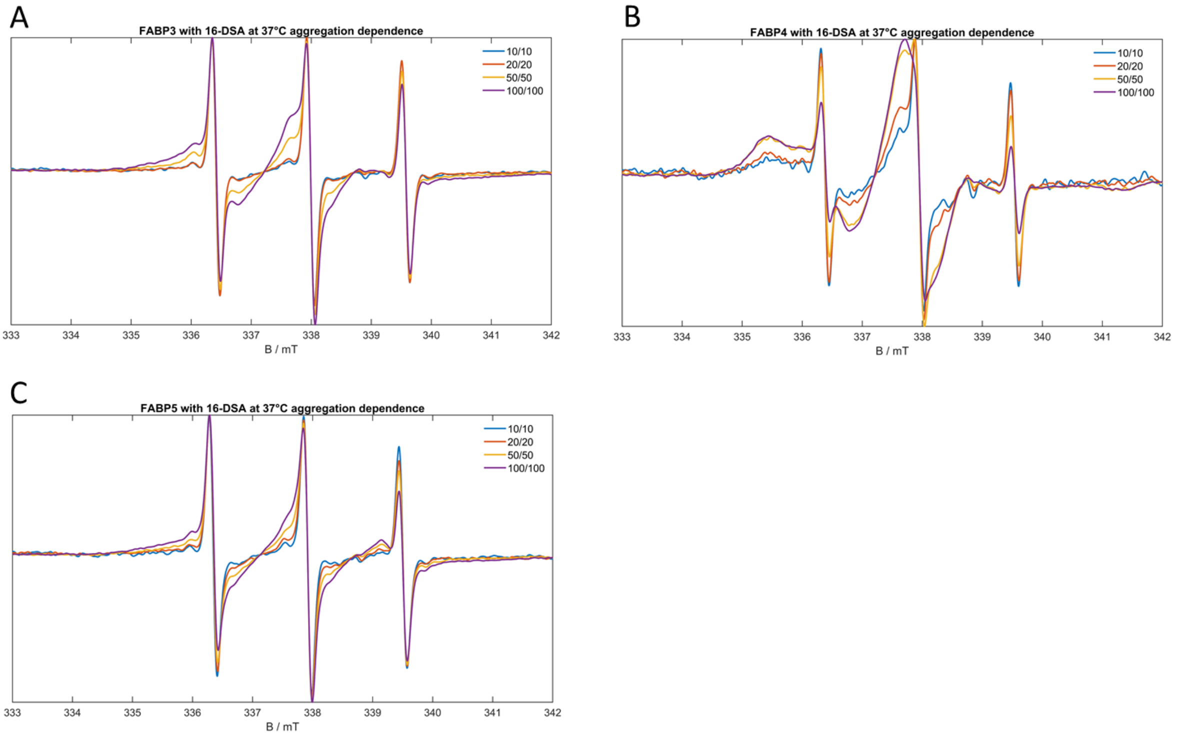


**Figure S16**: Intensity-normalized CW EPR spectra of 10/10, 20/20, 50/50 and 100/100 µM 16-DSA with FABP3 (A), FABP4 (B) and FABP5 (C) in HEPES at pH 7.5 and 37 °C. The linewidth of the spectra was increased artificially.

*Blue native PAGE and SDS PAGE*

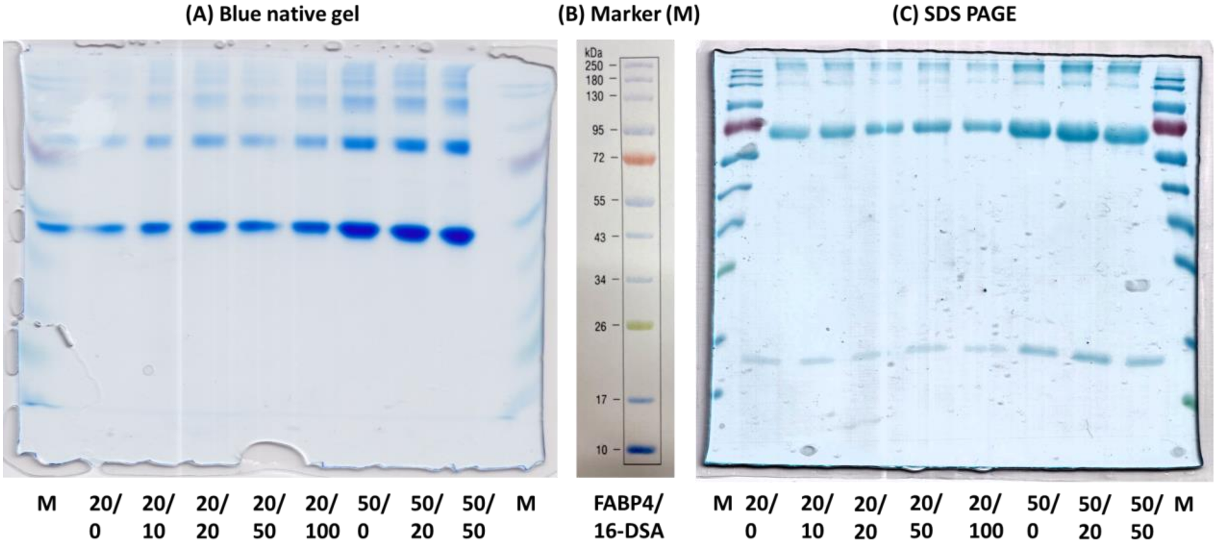


**Figure S17**: Blue native gel (A), molecular size marker (M) and SDS PAGE of FABP4/16-DSA samples 20/0, 20/10, 20/20, 20/50, 20/100, 50/0, 50/20 and 50/50.

Gel preparation: The 10 % native separating gel was prepared with 4.11 mL $H_2O$, 2.5 mL 1.5 Tris at pH 8.8, 3.33 mL Bis-Acrylamide (30 %), 10 % APS. The stacking gel was prepared from 6.2 mL $H_2O$, 2.5 mL 0.5 Tris at pH 6.8, 1.33 mL Bis-Acrylamide (30 %), 50 µL APS (10 %) and 5 µL TEMED. For 5x running buffer (1 L) 15 g Tris and 72 g glycine were dissolved in water and set to pH 8.3. For 5x native loading dye (pH 8.5) 2.5 mL Tris buffer (1 M) at pH 6.8 were mixed with 4 mL glycerol (100 %) and 0.005 g bromophenol blue.

*Supporting DLS particle radius distributions*

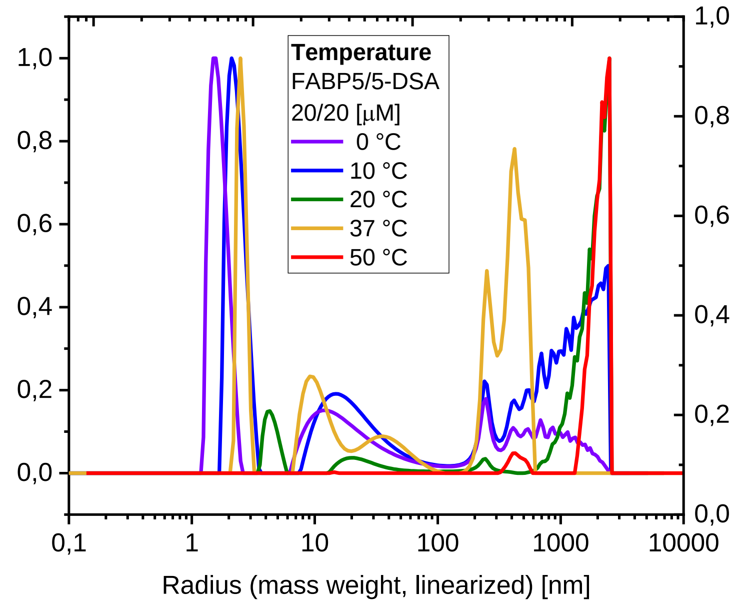


**Figure S18**: Particle radius distributions of 20 µM FABP5 with 20 µM 5-DSA in buffer at various temperatures.

*Temperature-dependent changes of $\tau_{c,iso}$ and $A'_{zz}$*

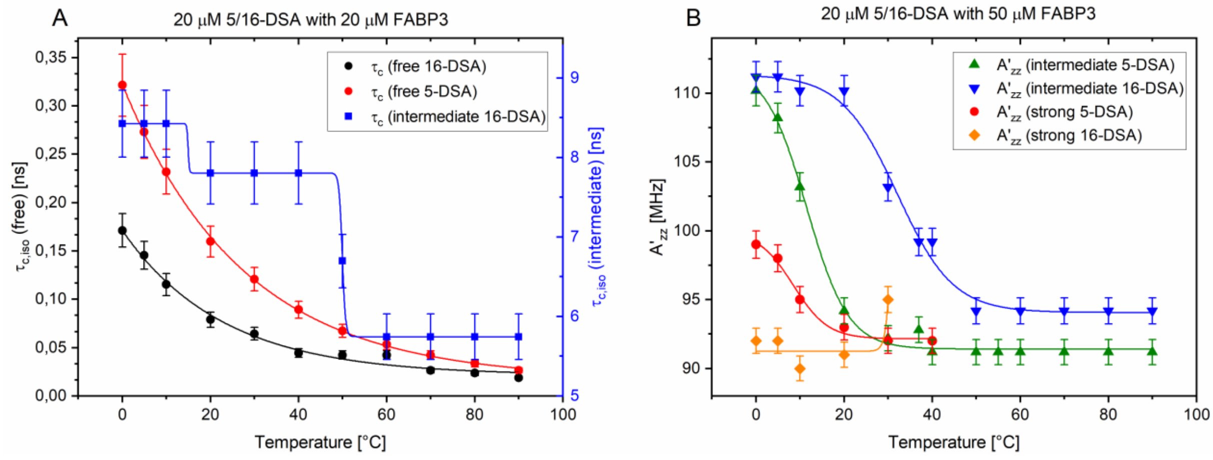

**Figure S19**: Temperature dependencies of $\tau_{c,iso}$ (A) and $A'_{zz}$ (B) from 5/16-DSA with FABP3. In this plot $A'_{zz}$ was used instead of $a_{iso}$ since only this tensor value was changed, the interpretation of both values can be seen as equivalent.

## *Docking simulation of FABP3 with 16-DSA*

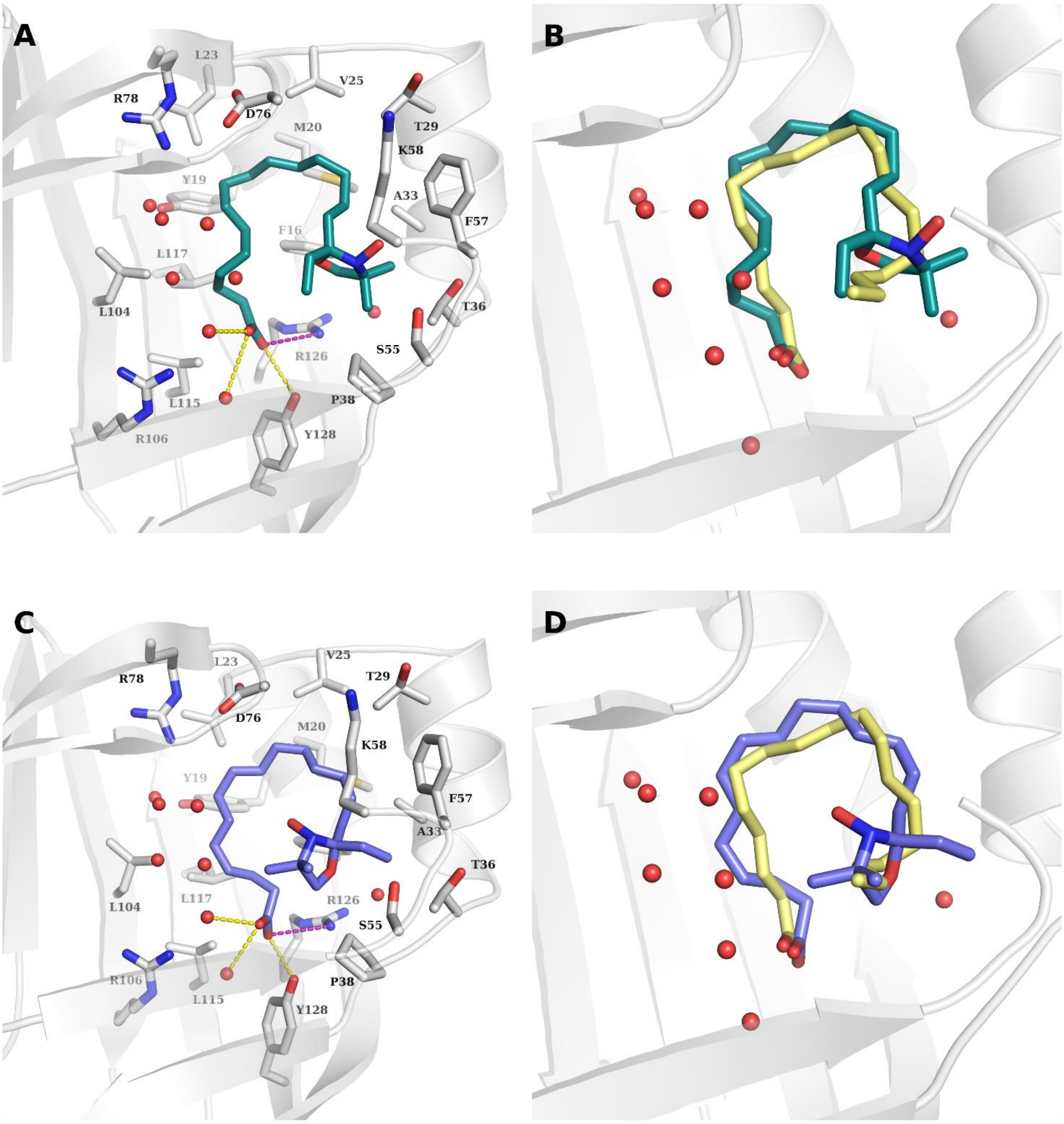


**Figure S20**: Predicted binding modes of R- and S-16-DSA in the binding cavity of FABP3 (PDB ID: 4WBK). A: Docking pose of R-16-DSA (teal sticks); B: overlaid with the experimentally determined binding mode of stearate (yellow sticks); C: Docking pose of S-16-DSA (purple-blue sticks); D: overlaid with the experimentally determined binding mode of stearate (yellow sticks). Relevant amino acids in the binding cavity are shown as white sticks and water molecules as red spheres. H-bond interactions are depicted as yellow dashed lines and salt-bridges as purple dashed lines.

*Bioinformatical analysis*

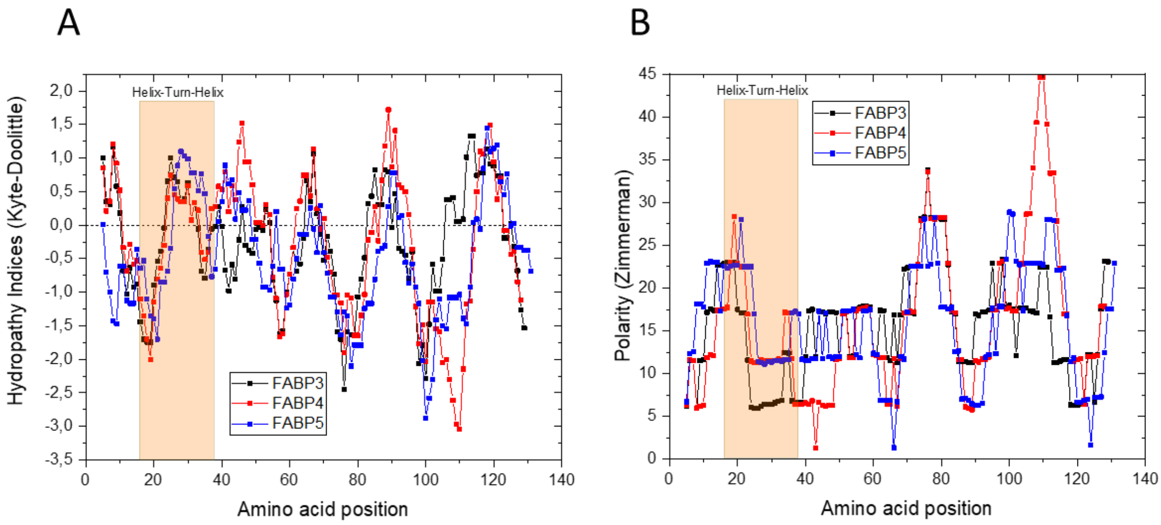


**Figure S21**: A: Hydropathy indices of the amino acid sequences of FABP3, FABP4 and FABP5 based on the scale of Kyte and Doolittle. B: Polarity of the sequences based on the scale of Zimmerman.

*Fit functions with parameter values for the concentration-dependent EPR binding curves*

**Table S4:** Fit functions for the concentration-dependent binding curves.

| System (component) | Fit function (name and mathematical expression with parameter values) |
|---|---|
| FABP3/5-DSA (T) | Hill: y = Vmax · $x^n/(k^n+x^n)$; Vmax = 98.766 ± 1.7; k = 0.475 ± 0.019; n = 1.898 ± 0.142 |
| | Bi Dose Response: double span = A2 - A1; double Section1 = span · p/(1+pow(10,(LOGx01-x) · h1)); double Section2 = span · (1-p)/(1+pow(10,(LOGx02-x) · h2)); y = A1 + Section1 +Section2; A1 = -25.049 ± 47.816; A2 = 100.111 ± 2.818; LOGx01 = 0.3528 ± 0.057; LOGx02 = 1.509 ± 10.914; h1 = 1.819 ± 0.451; h2 = 0.254 ± 0.476; p = 0.828 ± 0.527 |
| FABP3/5-DSA (I) | Dose Response (0-35 µM): y = A1 + (A2-A1)/(1 + $10^{((LOGx0-x)*p)}$); A1 = -22.128 ± 6.751; A2 = 87.196 ± 1.712; LOGx0 = 0.325 ± 0.032; p = 1.818 ± 0.195 |
| | Polynomial (35-200 µM): y = Intercept + B1 · $x^1$ + B2 · $x^2$; Intercept = 0.719; B1 = 4.941; B2 = -0.75 |
| FABP3/5-DSA (S) | Polynomial: y = Intercept + B1 · $x^1$ + B2 · $x^2$; Intercept = 98.633; B1 = 0.474; B2 = 1.888 |
| FABP3/16-DSA (T) | Hill: y = Vmax · $x^n/(k^n+x^n)$; Vmax = 100.399 ± 0.459; k = 0.535 ± 0.005; n = 2.057 ± 0,04 |

| | |
|---|---|
| FABP3/16-DSA (I) | Hill (0-50 µM): $y = Vmax \cdot x^n/(k^n+x^n)$; Vmax = 101.88 ± 0.634; k = 0.545 ± 0.005; n = 1.993 ± 0.035<br>Polynomial (50-200 µM): $y = Intercept + B1 \cdot x^1 + B2 \cdot x^2$; Intercept = 88.5; B1 = 5.26; B2 = -0.68 |
| FABP3/16-DSA (S) | Polynomial: $y = Intercept + B1 \cdot x^1 + B2 \cdot x^2$; Intercept = 0.875 ± 0.429; B1 = -1.675 ± 0.329; B2 = 0.417 ± 0.032 |
| FABP4/5-DSA (T) | Bi Hill: $y = Pm/(1+(Ka/x)^{Ha})/(1+(x/Ki)^{Hi})$; Pm = 126.51 ± 898.736; Ka = 2.328 ± 12.462; Ki = 18.0 ± 129.271; Ha = 1.081 ± 0.73; Hi = 4.721 ± 21.08<br>Double Boltzmann: y = y0+A·(frac/(1+exp((x-x01)/k1))+(1-frac)/(1+exp((x-x02)/k2))); y0 = -9.194 ± 4.391; A = 107.802 ± 4.512; frac = 0.608 ± 0.059; x01= 0.658 ± 0.046; x02 = 4.088 ± 0.405; k1 = -0.36 ± 0.063; k2 = -0.699 ± 0.231 |
| FABP4/5-DSA (I) | Hill (0-50 µM): $y = Vmax \cdot x^n/(k^n+x^n)$; Vmax = 66.431 ± 2.207; k = 0.852 ± 0.034; n = 2.115 ± 0.155<br>Polynomial (50-200 µM): $y = Intercept + B1 \cdot x^1 + B2 \cdot x^2$; Intercept = 40.9; B1 = 9.7; B2 = -0.824 |
| FABP4/5-DSA (S) | Linear (0-50 µM): y = 0<br>Polynomial (50-200 µM): $y = Intercept + B1 \cdot x^1 + B2 \cdot x^2$; Intercept = -27.233; B1 = 12.18; B2 = -0.515 |
| FABP4/16-DSA (T) | Hill: $y = Vmax \cdot x^n/(k^n+x^n)$; Vmax = 92.29 ± 2.876; k = 0.797 ± 0.047; n = 1.454 ± 0.159 |
| FABP4/16-DSA (I) | Bi Hill: $y = Pm/(1+(Ka/x)^{Ha})/(1+(x/Ki)^{Hi})$; Pm = 280.934 ± 32317.052; Ka = 2.287 ± 119.725; Ki = 3.114 ± 849.731; Ha = 1.163 ± 13.831; Hi = 0.672 ± 18.443 |
| FABP4/16-DSA (S) | Polynomial: $y = Intercept + B1 \cdot x^1 + B2 \cdot x^2$; Intercept = 0.855 ± 0.458; B1 = -1.36 ± 0.33; B2 = 0.284 ± 0.032 |
| FABP5/5-DSA (T) | Hill: $y = Vmax \cdot x^n/(k^n+x^n)$; Vmax = 96.295 ± 0.907; k = 0.352 ± 0.009; n = 1.109 ± 0.041<br>Double Boltzmann: y = y0+A·(frac/(1+exp((x-x01)/k1))+(1-frac)/(1+exp((x-x02)/k2))); y0 = -902.716 ± 4007.407; A = 997.5231 ± 4008.444; frac = 0.777 ± 5.784; x01 = -0.739 ± 2.804; x02 = -4.238 ± 69.995; k1 = -0.314 ± 0.222; k2 = -2.153 ± 2.718 |
| FABP5/5-DSA (I) | Bi Hill (0-50 µM): $y = Pm/(1+(Ka/x)^{Ha})/(1+(x/Ki)^{Hi})$; Pm = 305.375 ± 118504.425; Ka = 1.356 ± 552.543; Ki = 1.232 ± 356.137; Ha = 1.046 ± 11.527; Hi = 1.14 ± 22.032<br>Polynomial (50-200 µM): $y = Intercept + B1 \cdot x^1 + B2 \cdot x^2$; Intercept = 55.667; B1 = 3.16; B2 = -0.171 |

| | |
|---|---|
| FABP5/5-DSA (S) | Boltzmann: y = A2 + (A1-A2)/(1 + exp((x-x0)/dx)); A1 = -0.321 ± 0.394; A2 = 23.893 ± 0.43; x0 = 1.614 ± 0.028; dx = 0,219 ± 0.025 |
| FABP5/16-DSA (T) | Hill: $y = Vmax \cdot x^n/(k^n+x^n)$; Vmax = 98.288 ± 1.318; k = 0.315 ± 0.011; n = 0.852 ± 0.04 |
| FABP5/16-DSA (I) | Bi Hill (0-50 µM): $y = Pm/(1+(Ka/x)^{Ha})/(1+(x/Ki)^{Hi})$; Pm = 96.194 ± 45.092; Ka = 0.3 ± 0.093; Ki = 3.876 ± 1.6; Ha = 0.905 ± 0.603; Hi = 3.565 ± 2.053<br>Polynomial (50-200 µM): $y = Intercept + B1 \cdot x^1 + B2 \cdot x^2$; Intercept = 61.4; B1 = 4.02; B2 = -0.28 |
| FABP5/16-DSA (S) | Boltzmann: y = A2 + (A1-A2)/(1 + exp((x-x0)/dx)); A1 = -0.128 ± 0.488; A2 = 17.521 ± 0.768; x0 = 2.047 ± 0.085; dx = 0.235 ± 0.0528 |
